# Supplementary material for: Intercellular transfer of cancer cell invasiveness via endosome-mediated protease shedding
Source: Nat Commun. 2024 Feb 10;15:1277. doi: 10.1038/s41467-024-45558-8 (PMC10858897; doi:10.1038/s41467-024-45558-8)
Supplement: Supplementary file 1 — Supplementary Information [file 41467_2024_45558_MOESM1_ESM.pdf]

Supplementary Information

Intercellular transfer of cancer cell invasiveness via endosome-mediated protease shedding

Wenzel et al. 2024

Nature Communications

Supplementary Figure 1

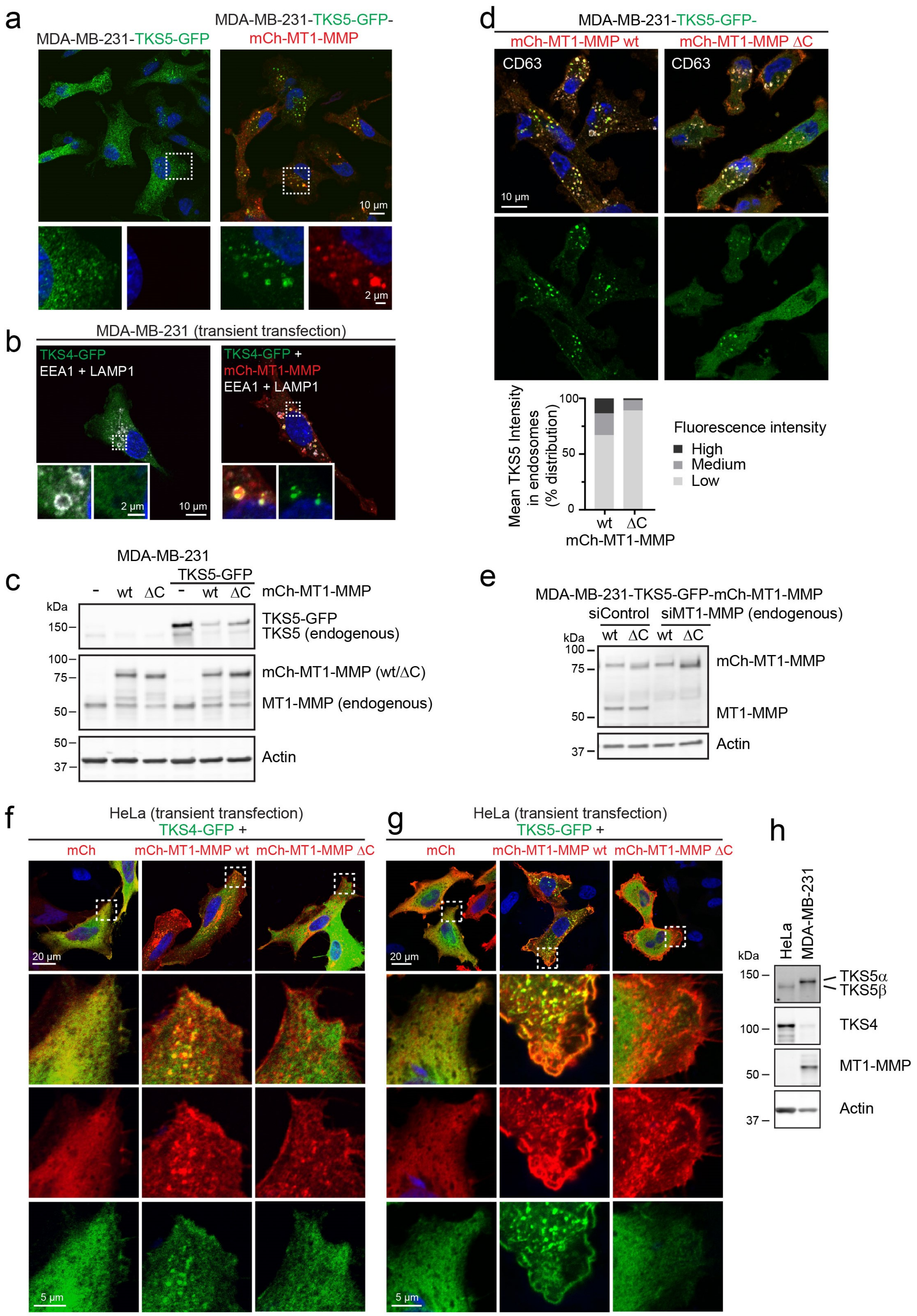

Supplementary Fig. 1 Endosome recruitment of TKS4/5 depends on the cytosolic tail of MT1-MMP (related to Fig. 1)

a) Confocal images of MDA-MB-231 cells stably expressing TKS5-GFP with or without stable expression of mCh-MT1-MMP. In the presence of mCh-MT1-MMP the TKS5-GFP localisation to endosomes is more pronounced. Representative of 5 confocal images per condition.

b) Confocal images of MDA-MB-231 cells transiently transfected with TKS4-GFP with or without mCh-MT1-MMP showing co-occurrence of mCh-MT1-MMP and TKS4-GFP at endosomes. Endosomes were stained with a mixture of EEA1 and LAMP1 antibodies. In the presence of mCh-MT1-MMP the TKS4-GFP localisation to endosomes is more pronounced. Representative of 10 confocal images per condition.

c) Characterization of stable cell lines used in this study. WB using lysates from MDA-MB-231 cell lines stably expressing TKS5-GFP and/or mCh-MT1-MMP constructs as indicated showing the expression levels of endogenous and exogenous TKS5 and MT1-MMP proteins. Representative of 2 WB.

d) MDA-MB-231 cells stably expressing TKS5-GFP in combination with either mCh-MT1-MMP wt or ΔC were fixed and immunostained with antibodies against TKS5, CD63 and mCh and analysed by confocal microscopy. The mean TKS5 intensity in CD63 and mCh-MT1-MMP co-positive dots was quantified automatically using Nikon NIS-Elements software. The graph represents the proportion of endosomes (% of total number of endosomes) with high, medium and low TKS5-GFP fluorescence intensities. Total number of cells: wt, 175; ΔC, 229. Total number of endosomes: wt, 894; ΔC, 807, from three independent coverslips.

e) MDA-MB-231-TKS5-GFP-mCh-MT1-MMP wt and ΔC cells were depleted for endogenous MT1-MMP by siRNA transfection and the efficiency of the depletion analysed by WB. Related to Fig. 1e. Representative of 3 WB.

f) HeLa cells were co-transfected with TKS4-GFP and mCh or MT1-MMP wt or ΔC, stained with antibodies against GFP and mCh and analysed by confocal microscopy. Representative of at least 5 images per condition.

g) HeLa cells were co-transfected with TKS5-GFP and mCh or MT1-MMP wt or ΔC, stained with antibodies against GFP and mCh and analysed by confocal microscopy. Representative of 8 images per condition.

h) Cell lysate from HeLa and MDA-MB-231 cell lines was subjected to WB analysis to assess the expression of TKS4, TKS5 and MT1-MMP. Note that MDA-MB-231 cells express MT1-MMP and TKS5α, whereas HeLa cells are negative for MT1-MMP and express a shorter isoform of TKS5, TKS5β, lacking the PX domain [58]. Both cell lines express TKS4. Representative of 2 WB.

Supplementary Figure 2

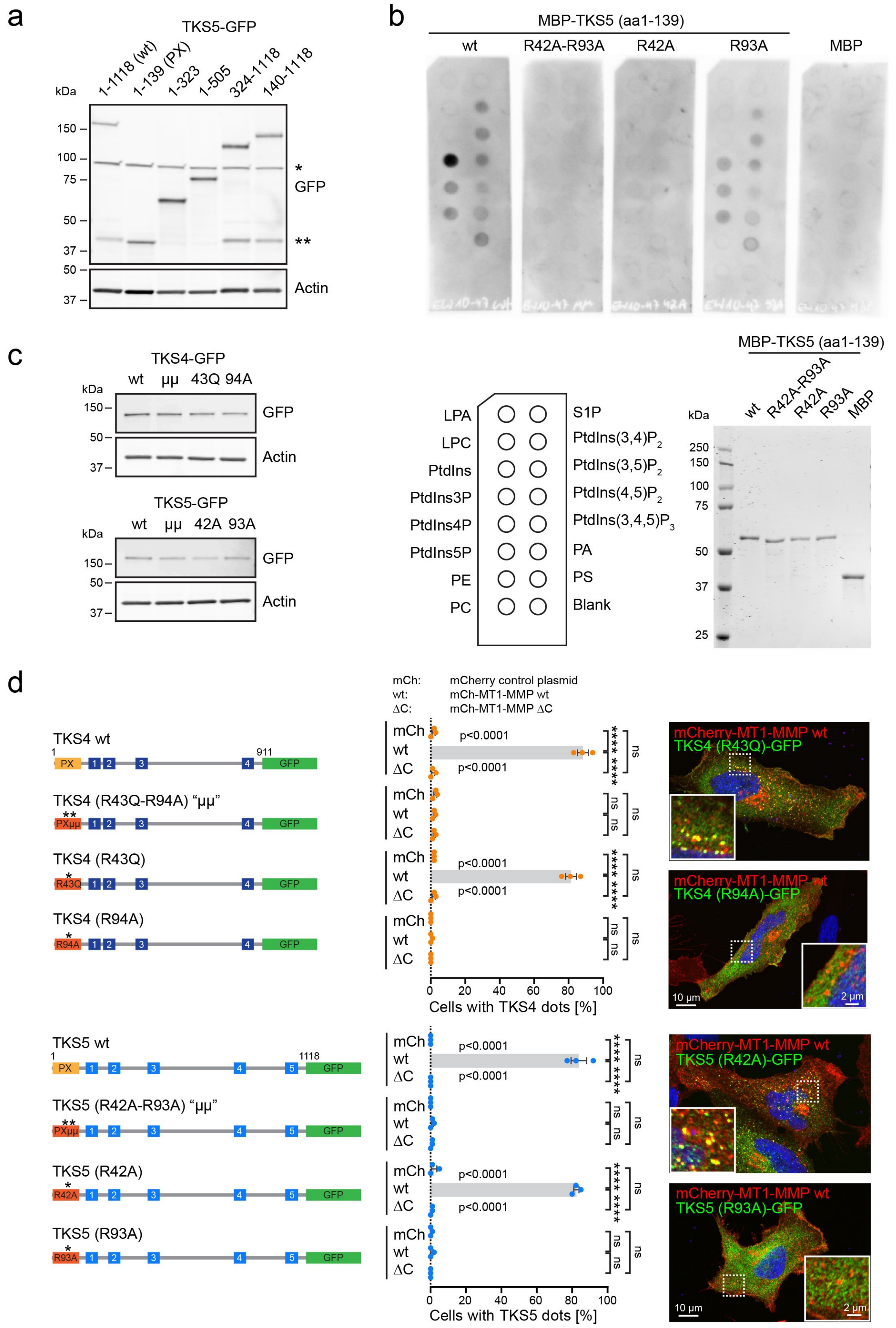

Supplementary Fig. 2 A functional PX domain of TKS4/5 is required for their endosome localisation (related to Fig. 2)

a) WB showing the expression of the TKS5-GFP deletion constructs used in this study. HeLa cells were transfected with the indicated plasmids. 24 to 48 hours after transfection, whole cell lysates were made and analysed by WB. * unspecific band, ** possibly alternative translation start using an ATG between the CC domain and the SH3#5 of TKS5. Representative of 2 WB.

b) Protein-lipid overlay assay: Purified MBP-fusions of the TKS5-PX domain as wt, R42A, R93A or double mutant were tested for lipid interactions using PIP strips^{TM}. Representative of 2 independent experiments. A Coomassie gel shows the purity and molecular size of the purified protein and ensures application of equal amounts to the PIP strips. LPA, lysophosphatidic acid; LPC, lysophosphocholine; PtdIns, phosphoinositide phosphates; PE, phosphatidylethanolamine; PC, phosphatidylcholine; S1P, sphingosine-1-phosphate; PA, phosphatidic acid; PS, phosphatidylserine.

c) WB showing the expression of the TKS4- and TKS5-GFP PX mutant constructs used in this study. HeLa cells were transfected with the indicated plasmids. 24 to 48 hours after transfection, whole cell lysates were made and analysed by WB. Representative of 2 WB.

d) HeLa cells were transfected with mCh or mCh-MT1-MMP wt or ΔC and cotransfected with the indicated TKS4- or TKS5-GFP constructs. The localisation of the TKS4/5 constructs was scored by fluorescence microscopy and example images with and without endosomal recruitment are displayed. The graphs show the percentage of cells with a dot-like localisation of the respective construct as mean +/- SEM from n=3 independent experiments. At least 100 cells were counted per condition and experiment. One-way ANOVA of each individual construct set of 3 conditions with Tukey's multiple comparisons test.

Supplementary Figure 3

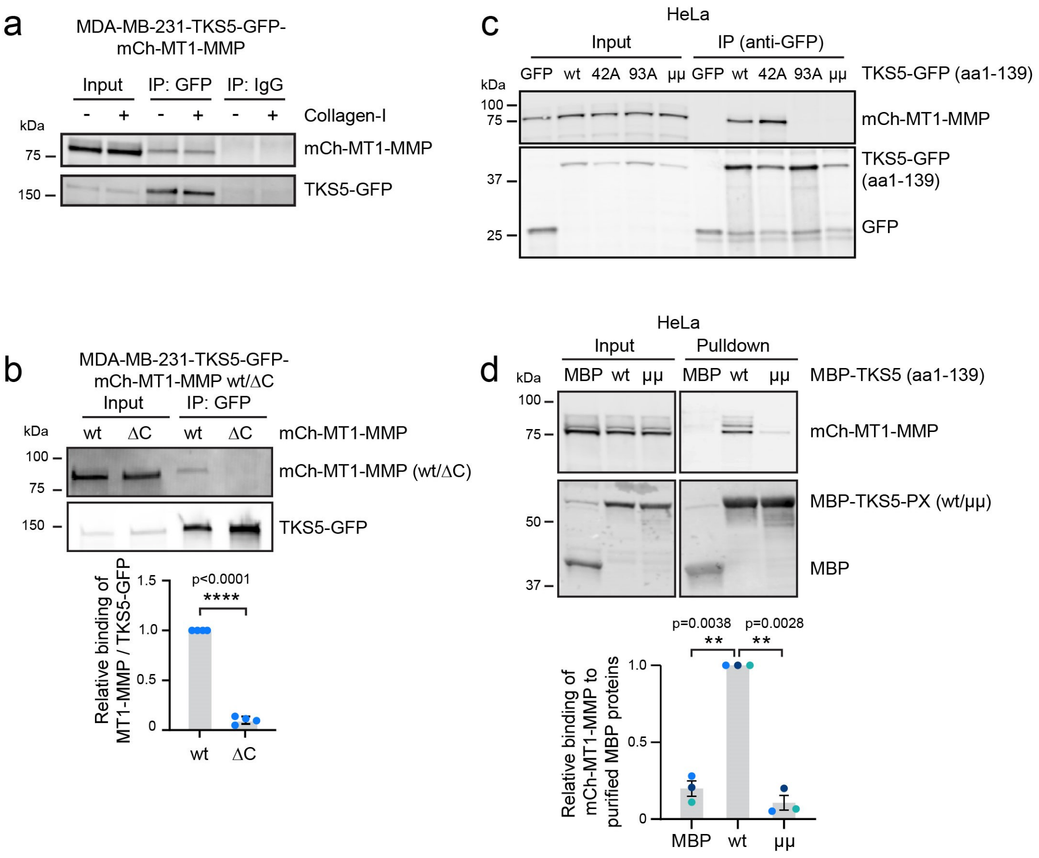

Supplementary Fig. 3 A functional PX domain of TKS5 interacts with the C-terminus of MT1-MMP (related to Fig. 2)

a) Co-immunoprecipitation experiment of mCh-MT1-MMP and TKS5-GFP in cells grown in the presence or absence of collagen-I. MDA-MB-231-TKS5-GFP-mCh-MT1-MMP cells were seeded directly in plastic cell culture dishes or on a layer of collagen-I. Cells were lysed and immunoprecipitation was performed using an anti-GFP antibody or mouse IgG as a negative control. mCh-MT1-MMP interacts with TKS5-GFP under both conditions. Representative of 2 WB.

b) MDA-MB-231-TKS5-GFP-mCh-MT1-MMP wt and ΔC cells were depleted for endogenous MT1-MMP by siRNA transfection. Protein lysates were used for immunoprecipitations using an antibody against GFP. The graph shows the relative intensities of co-immunoprecipitated mCh-MT1-MMP wt or ΔC as mean +/- SEM of n=4 independent experiments. One-sample two-sided t-test.

c) Co-immunoprecipitation experiment of PX domain mutants of TKS5-GFP and mCh-MT1-MMP in HeLa cells. HeLa cells were co-transfected with mCh-MT1-MMP and either GFP alone, TKS5-GFP PX wt, TKS5-GFP PX R42A, TKS5-GFP PX R93A or TKS5-GFP PX double mutant (μμ). Immunoprecipitation demonstrated that the mutants R93A and μμ were unable to bind mCh-MT1-MMP. GFP alone serves as negative control. Representative of 3 experiments.

d) Mannose-binding-protein (MBP) pulldown experiment. Purified MBP-TKS5 wt, but not MBP-TKS5 μμ binds to mCh-MT1-MMP from the lysate of transfected HeLa cells. Purified MBP alone serves as a negative control. Graph shows the mean intensity of the MT1-MMP band quantified from n=3 independent experiments +/- SEM. One-sample two-sided t-test.

Supplementary Figure 4

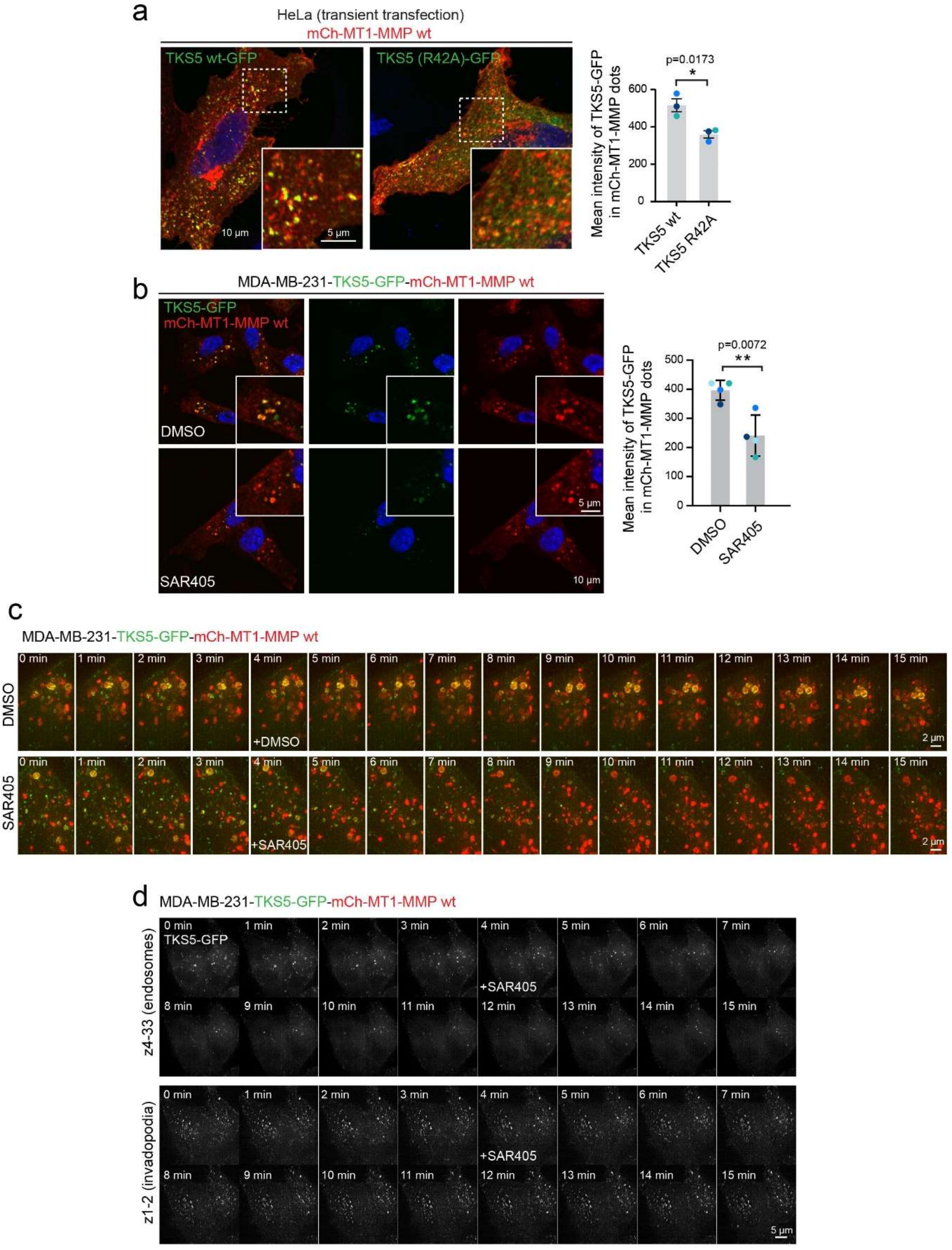

Supplementary Fig. 4 TKS4/5 recruitment to endosomes is PtdIns3P-dependent (related to Fig. 2)

a) HeLa cells were co-transfected with mCh-MT1-MMP wt and TKS5-GFP wt or R42A, stained with antibodies against GFP and mCh and analysed by confocal microscopy. The graph represents the mean fluorescence intensity of TKS5-GFP in mCh-MT1-MMP dots analysed automatically using Nikon NIS-Elements software. Error bars denote mean +/- SEM from n=3 independent experiments indicated by colored dots. In total, 68 cells were analysed per condition. Unpaired two-sided t-test.

b) MDA-MB-231-TKS5-GFP-mCh-MT1-MMP cells were treated with DMSO or 6 µM SAR405 for 15 min, stained with antibodies against GFP and mCh and analysed by confocal microscopy. The graph represents the mean fluorescence intensity of TKS5-GFP in mCh-MT1-MMP dots, analysed automatically using Nikon NIS-Elements software. Error bars denote mean +/- SD from n=4 independent experiments indicated by colored dots. In total 233 (DMSO) and 231 (SAR405) cells were analysed. Unpaired two-sided t-test.

c) Live-cell imaging of MDA-MB-231-TKS5-GFP-mCh-MT1-MMP cells. Shown are image stills from movie 1. DMSO or SAR405 (6 µM end concentration) were added 3 minutes after starting the image acquisition. Displayed is a maximum intensity projection comprising the whole image stack of one representative cell. TKS5-GFP dissociates from endosomes upon addition of SAR405. DMSO is used as a control. In total, 3 DMSO/SAR405 experiments were recorded, imaging 6 ROIs per experiment, each ROI comprising several cells.

d) Image stills from movie 2, to illustrate that endosomal, but not invadopodial TKS5-GFP is affected by SAR405 addition. Example from the same experimental dataset as in c). Upper panel: Maximum intensity projection of the apical part of a cell (z slices 4-33) comprising endosomes and cytosol. TKS5-GFP dissociates from endosomes upon addition of SAR405. Lower panel: Maximum intensity projection of the two most basal slices (z slices 1-2) from the image stack of the same cell, comprising invadopodia. Here, TKS5-GFP is unaffected by the addition of SAR405.

Supplementary Figure 5

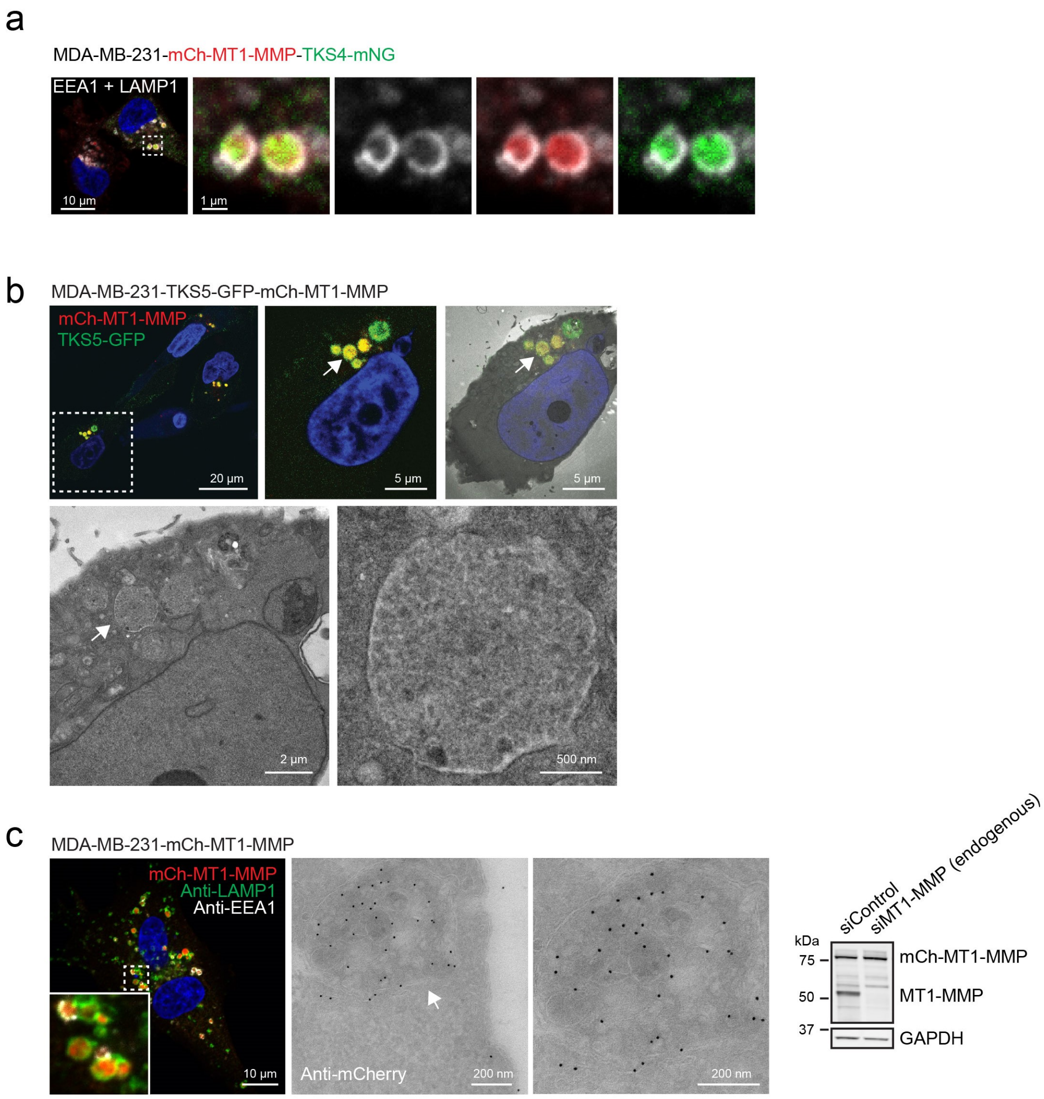

Supplementary Fig. 5 TKS4/5 and MT1-MMP localise in multivesicular endosomes (related to Fig. 3)

a) MDA-MB-231-mCh-MT1-MMP-TKS4-mNeonGreen cells were grown on cover slips, fixed and immunostained with antibodies against mNG, mCh and EEA1 + LAMP1, and analysed by confocal microcopy. TKS4-mNG and mCh-MT1-MMP appear to localise inside endosomes. Representative of 5 confocal images.

b) Correlative light and electron microscopy analysis of MDA-MB-231-TKS5-GFP-mCh-MT1-MMP cells shows that endosomes positive for both mCh-MT1-MMP and TKS5-GFP (confocal images) are MVEs (EM images). The cells were fixed and stained with Hoechst for imaging by confocal microscopy before being processed for electron microscopy. Representative of 10 cells from 4 dishes.

c) Immuno-EM experiment to analyse the localisation of mCh-MT1-MMP. MDA-MB-231-mCh-MT1-MMP cells were depleted for endogenous MT1-MMP to be able to assess the trafficking and internalization of mCh-MT1-MMP without a potential contribution of endogenous MT1-MMP though dimerization. KD efficiency and cell morphology were controlled by IF staining and WB analysis, respectively. In parallel, samples were processed for immuno-EM (Tokayasu) as explained in the methods. mCh-MT1-MMP could be detected inside an MVE using an anti-mCh antibody (10 nm gold particles). The arrow points to the limiting membrane of the MVE, which is enlarged in the right panel. Representative of 46 MVEs from 4 independent experiments and 4 WB.

Supplementary Figure 6

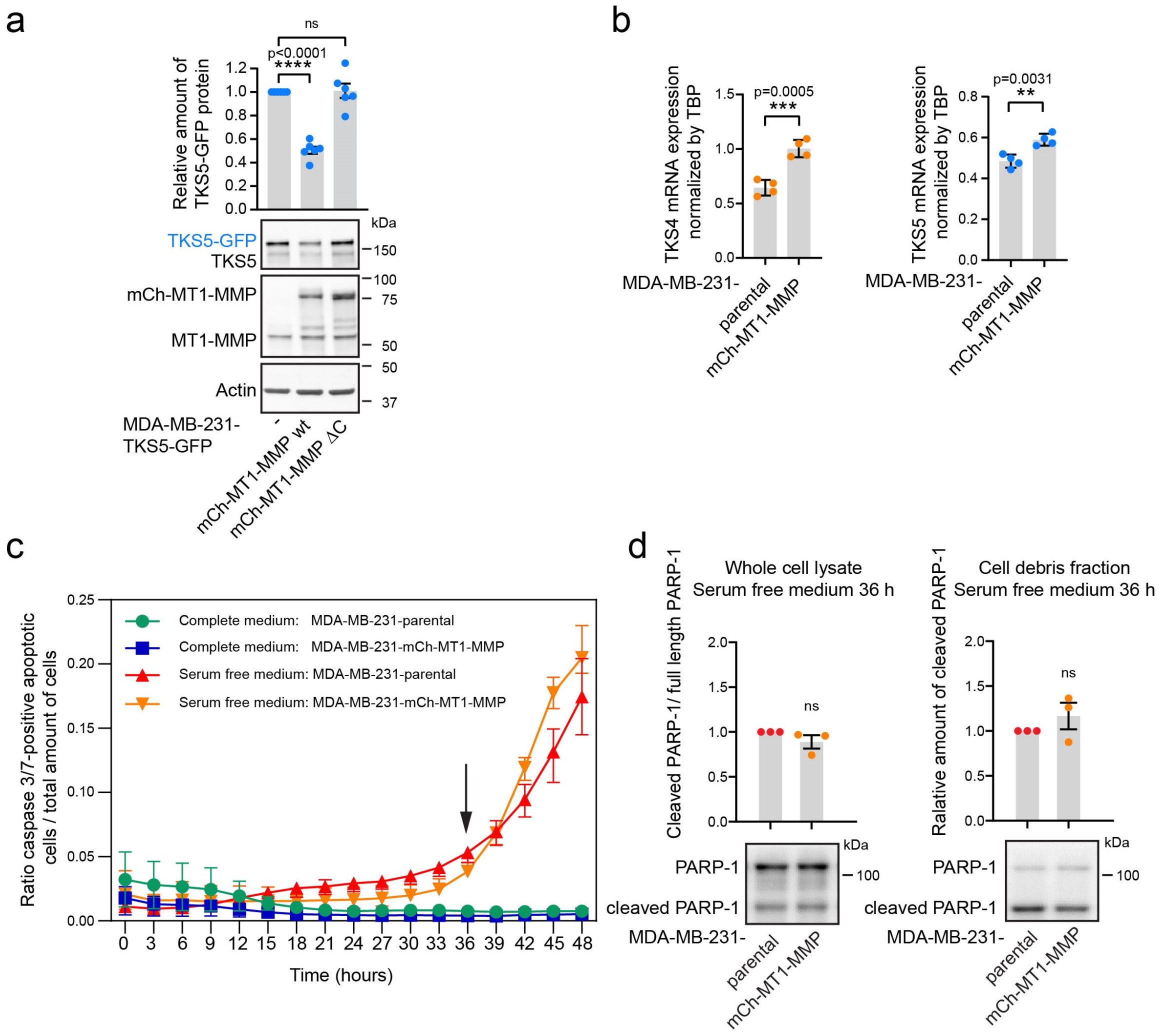

Supplementary Fig. 6 Levels of TKS4/5 and apoptotic activity in MT1-MMP expressing cells (related to Fig. 3)

a) Relative protein levels of TKS5-GFP quantified from cell lysates of MDA-MB-231-TKS5-GFP cells with or without stable expression of mCh-MT1-MMP wt or -ΔC. The graph represents mean +/-SEM. n=6 independent experiments. One-sample two-sided t-test.

b) mRNA levels of TKS4 or TKS5 quantified by qPCR from MDA-MB-231 cells with or without stable expression of mCh-MT1-MMP. 2 independent experiments with 2 replicates, mean +/- SD. Unpaired two-sided t-test.

c) Control experiment for Fig. 3c. Ratio of apoptotic cells/total amount of cells of indicated cell lines and treatments measured automatically by IncuCyte® using IncuCyte® Caspase-3/7 Dye for Apoptosis, normalized to cell confluency. Error bars denote mean +/- SD from 2-4 replicates from one experiment. Note that the level of apoptosis observed after serum withdrawal is similar for the two cell lines after 36 hours, which is the time point for EV isolation, controlling that the increased level of TKS4/5 in the EV fraction is not caused by increased amounts of apoptotic bodies in MDA-MB-231-mCh-MT1-MMP cells.

d) Control experiment for Fig. 3c. WB and quantifications of cleaved PARP-1/PARP-1 in whole cell lysate and apoptotic body enriched cell debris fraction from the indicated cell lines after 36 hours serum withdrawal from n=3 independent experiments. Error bars denote mean +/- SEM. One-sample two-sided t-test.

Supplementary Figure 7

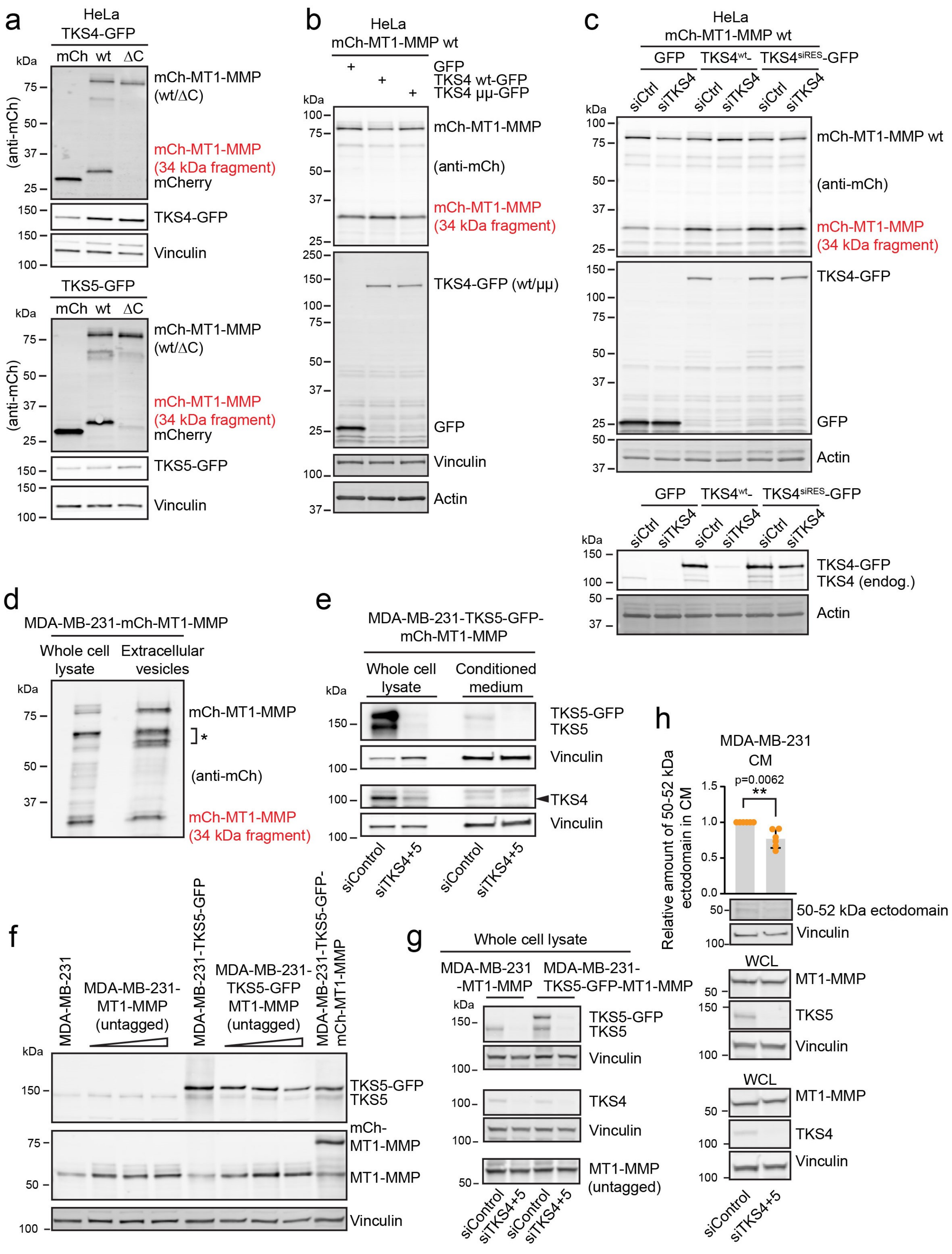

Supplementary Fig. 7 TKS4/5 promote cleavage of MT1-MMP and colocalise with ADAM15 (related to Fig. 4)

a) HeLa cells were transfected with the indicated constructs, lysed and subjected to WB. mCh-MT1-MMP wt, but not ΔC shows a 34 kDa fragment, detectable with an antibody directed against mCh. Representative for 2 (TKS4) and 3 (TKS5) WB.

b) WB representative for the dataset described and quantified in Fig. 4d.

c) WBs representative for the dataset described and quantified in Fig. 4e.

d) Whole cell lysate and extracellular vesicle fraction (100 000×g) from MDA-MB-231-mCh-MT1-MMP cells analysed by WB using anti-mCh. * MT1-MMP variants likely comprising processed forms of MT1-MMP and mCh-MT1-MMP (immature, posttranslationally modified or cleaved).

e) WB showing KD of TKS4 and TKS5 corresponding to Fig. 4f.

f) WB showing the expression level of TKS5-GFP and MT1-MMP in the indicated stable cell lines.

g) WB showing KD of TKS4 and TKS5 corresponding to Fig. 4g.

h) WB and quantification of the amount of MT1-MMP shed ectodomain in conditioned medium (CM) and whole cell lysates (WCL) from siRNA transfected MDA-MB-231 cells. Error bars denote mean +/- SD from n=6 independent experiments. One-sample two-sided t-test.

Supplementary Figure 8

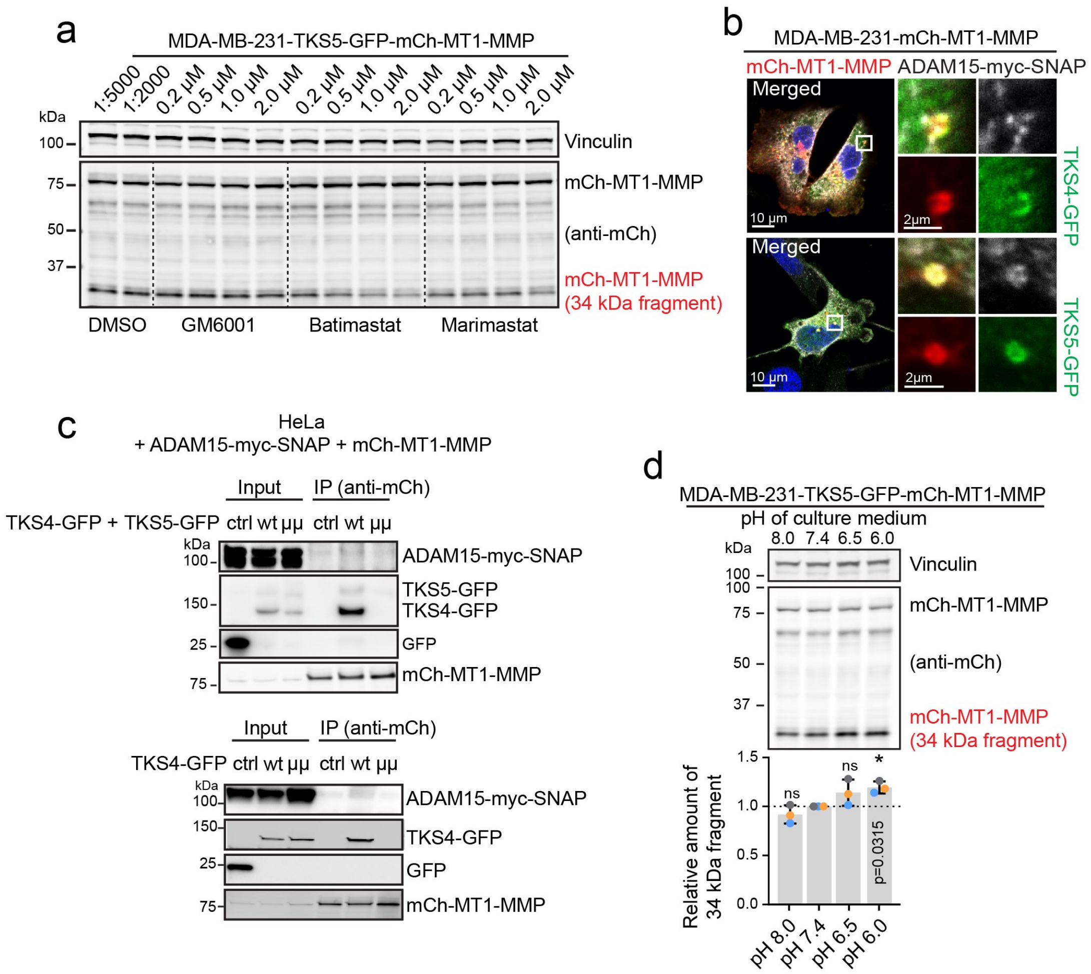

Supplementary Fig. 8 ADAM15 associates with TKS4/5 and MT1-MMP (related to Fig. 5)

a) MDA-MB-231-TKS5-GFP-mCh-MT1-MMP cells were treated with metalloproteinase inhibitors as indicated. After 24 hours of inhibitor treatment, protein lysates were made and analysed by WB. WB is representative for three independent experiments.

b) MDA-MB-231-mCh-MT1-MMP cells were transiently transfected with TKS4- or TKS5-GFP and ADAM15-SNAP for 24 hours, fixed in 3% FA, stained with anti-mCh, anti-GFP and anti-SNAP antibodies and analysed by confocal microscopy. Representative of 7 (TKS4) and 5 (TKS5) images.

c) Co-immunoprecipitation experiment using HeLa cells transfected with mCh-MT1-MMP, ADAM15-myc-SNAP and the indicated wt or µµ version of TKS4-GFP and TKS5-GFP or GFP (ctrl). ADAM15-myc-SNAP preferentially co-immunoprecipitates with mCh-MT1-MMP in the presence of wt, but not mutated TKS4/5-GFP or GFP alone. Quantifications are shown in Fig. 5c.

d) The pH of the cell culture medium of MDA-MB-231-TKS5-GFP-mCh-MT1-MMP cells was adjusted to the indicated values using HCl_{aq}. 20 hours later, total cell lysates were analysed by WB. The graph shows the quantification of the 34 kDa cleavage product of MT1-MMP. Data is from n=3 independent experiments mean +/- SD. One sample two-sided t-test.

Supplementary Figure 9

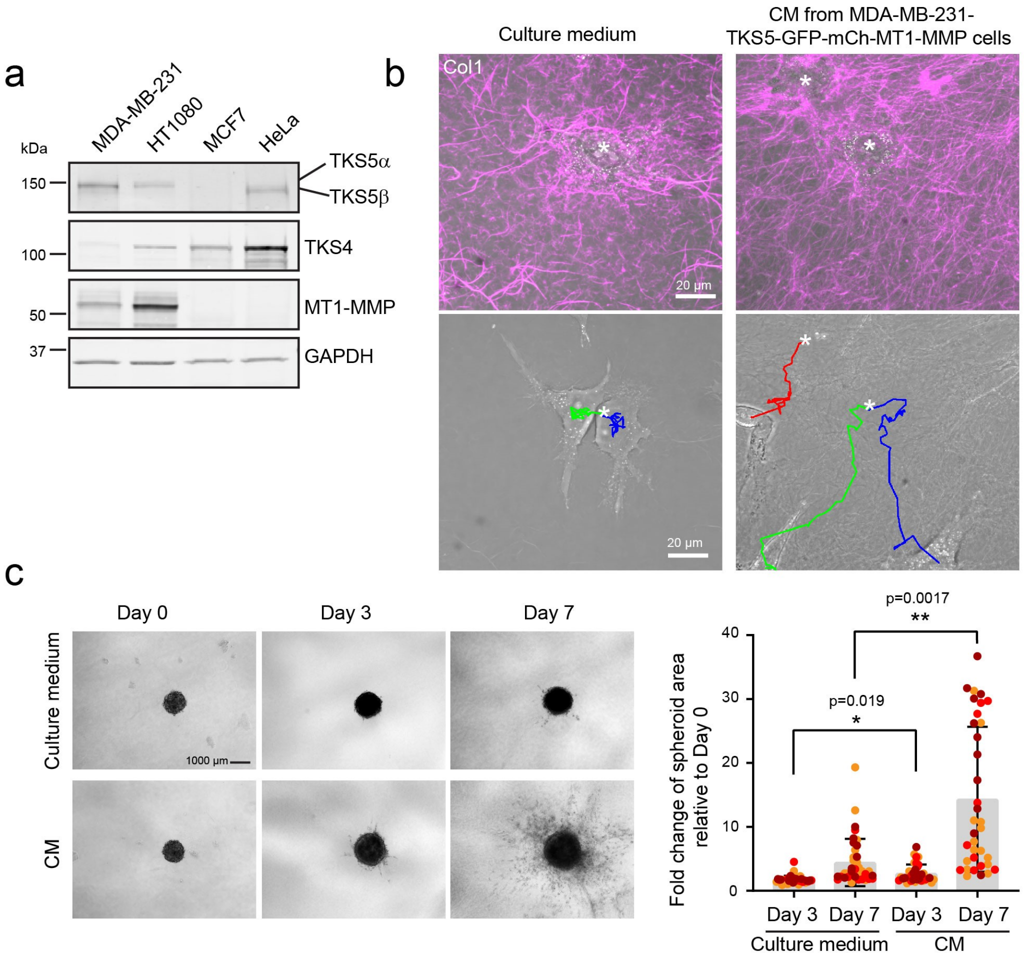

Supplementary Fig. 9 HeLa cells become invasive after treatment with conditioned medium from MDA-MB-231-TKS5-GFP-mCh-MT1-MMP cells (related to Fig. 6)

a) WB showing the expression levels of MT1-MMP, TKS4 and TKS5 in MDA-MB-231, HT1080, MCF7 and HeLa cells. Representative of 2 WB.

b) HeLa cells were embedded in 3D type I collagen gel (magenta) in 8 well glass bottom slides and incubated with either regular cell culture medium or conditioned medium (CM) from MDA-MB-231-TKS5-GFP-mCh-MT1-MMP cells and analysed by real-time spinning disk confocal microscopy for up to 24 hours. The time-lapse acquisition was started 60 min after adding the medium. Images were taken every 20 min. The upper panel shows the first frames from movie 3. Cells marked with an * were tracked manually using ImageJ/Fiji, and the cell tracks are shown in the lower panel (last frame of the movie). Note that the cells divide during the imaging. Corresponding to the dataset presented in Fig. 6d.

c) Multicellular spheroids from HeLa cells were embedded in type I collagen and incubated with either culture medium or CM from MDA-MB-231-TKS5-GFP-mCh-MT1-MMP cells, and were allowed to invade for 7 days. Phase contrast images show spheroids from day 0, 3 and 7. Graph represents fold increase of spheroid area on day 3 and 7 relative to its own spheroid area on day 0. In total, n=38 spheroids for regular full medium, n=35 for CM, from n=3 independent experiments, mean +/- SD. The different experiments are color coded. Kruskal-Wallis test with Dunn’s multiple comparisons test.

Supplementary Figure 10

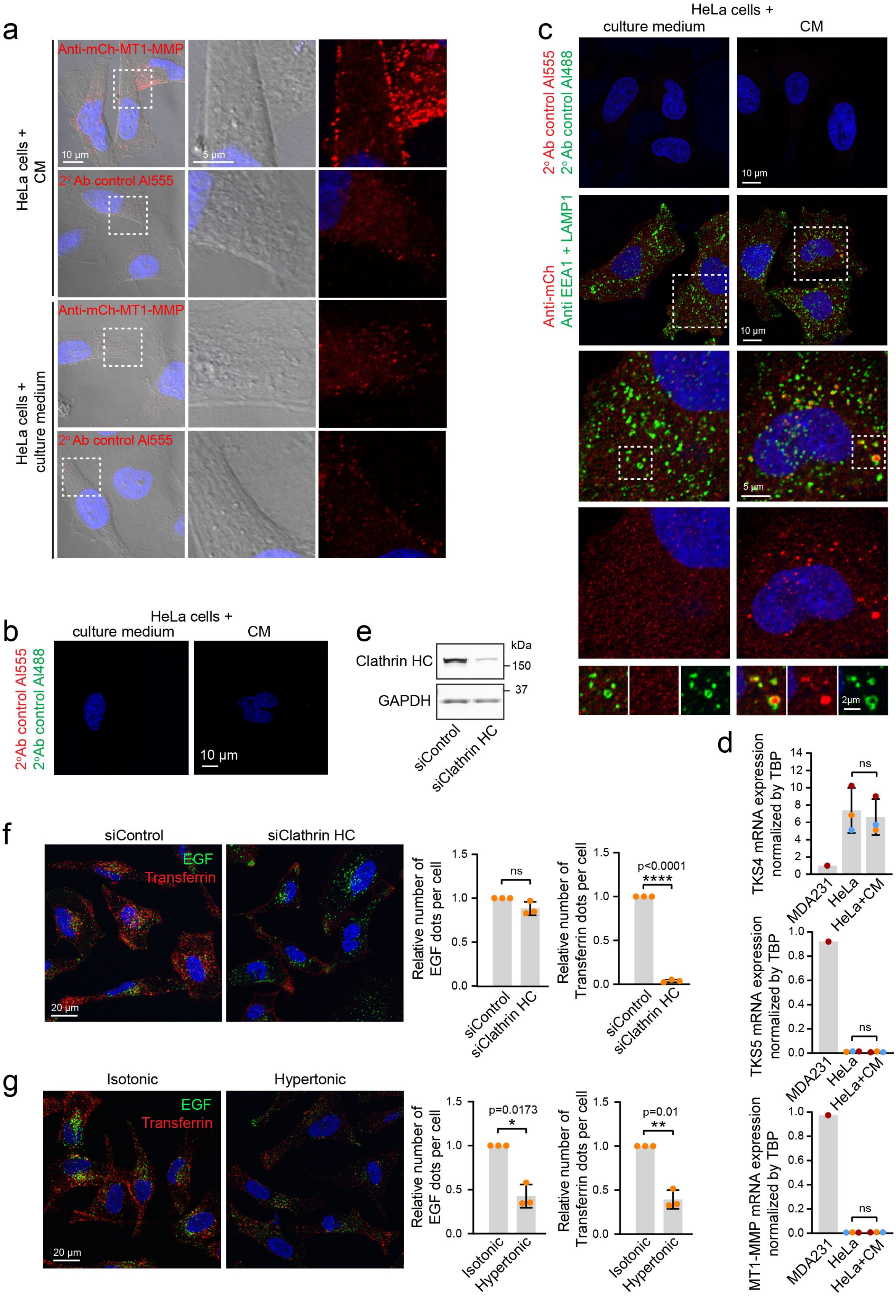

Supplementary Fig. 10 mCh-MT1-MMP from conditioned medium of MDA-MB-231 cells is detected in HeLa cells (related to Fig. 7)

a) Control experiments corresponding to Fig. 7c. HeLa cells were incubated with culture medium or conditioned medium (CM) from MDA-MB-231-TKS5-GFP-mCh-MT1-MMP cells for 2 hours, fixed in 3% FA and stained with secondary antibodies only or antibodies against mCh as indicated, in the absence of detergent. mCh-MT1-MMP was detected with an anti-mCh antibody on the cells treated with the CM only.

b) Secondary antibody only control corresponding to Fig. 7d, to show that the signal from TKS5-GFP or unboosted mCh-MT1-MMP from the CM is not visible under the imaging conditions used.

c) Control experiments corresponding to Fig. 7e. HeLa cells were treated with culture medium or CM from MDA-MB-231-TKS5-GFP-mCh-MT1-MMP cells for 2 hours, fixed in 3% FA, permeabilized with 0.05% saponin, and stained with secondary antibodies only (upper) or antibodies against mCh and endosomes (anti-LAMP1 and anti-EEA) as indicated. Note that under the chosen imaging conditions, the signal from any TKS5-GFP or mCh-MT1-MMP from the CM is not visible. Therefore, it was possible to detect endosomes using Al488 secondary antibodies. mCh-MT1-MMP was detected with an anti-mCh antibody in the cells treated with the CM only.

d) mRNA levels of TKS4, TKS5 or MT1-MMP quantified by qPCR from MDA-MB-231 cells (one experiment) or HeLa cells (3 independent experiments) treated or not with CM from MDA-MB-231-TKS4-GFP-mCh-MT1-MMP cells. The graphs represent mean +/- SD. One-way ANOVA with Tukey's multiple comparisons test.

e) WB showing the KD efficiency following siRNA-mediated depletion of clathrin heavy chain (CHC), related to Fig. 7g.

f) Control experiment corresponding to Fig. 7g, showing that cellular uptake of transferrin is inhibited in Clathrin HC depleted cells. HeLa cells were incubated with CM from MDA-MB-231-TKS4-GFP-mCh-MT1-MMP cells and 50 ng/ml Alexa488EGF for 24 hours. The last 10 min, the cells were incubated with 25 µg/ml Alexa568transferrin, fixed and analysed by confocal microscopy. The graphs represent the relative number of fluorescent dots per cell quantified automatically using Nikon NIS-Elements software, mean +/- SD from n=3 independent experiments. Note that uptake of Alexa488EGF is not inhibited by CHC KD, due to the high dose of Alexa488EGF, which is internalized by clathrin independent endocytosis. In total >95 cells were analysed per condition. One sample two-sided t-test.

g) Control experiment corresponding to Fig. 7h, showing that cellular uptake of transferrin and EGF is inhibited in cells treated with hypertonic medium. HeLa cells were incubated with isotonic or hypertonic (addition of 100 mM NaCl) CM from MDA-MB-231-TKS4-GFP-mCh-MT1-MMP cells and 50 ng/ml Alexa488EGF for 24 hours. The last 10 min, the cells were incubated with 25 µg/ml Alexa568transferrin, fixed and analysed by confocal microscopy. The graphs represent the relative number of fluorescent dots per cell quantified automatically using Nikon NIS-Elements software, mean +/- SD from n=3 independent experiments. In total >80 cells were analysed per condition. One sample two-sided t-test.

Supplementary Figure 11

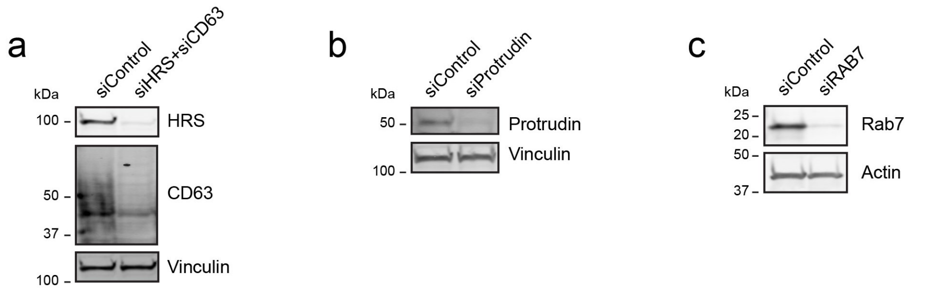

Supplementary Fig. 11 Knock down verification of HRS, CD63, Protrudin and RAB7 (related to Fig.8b-e)

a) WB showing the KD efficiency following siRNA-mediated co-depletion of HRS and CD63 in HeLa cells.

b) WB showing the KD efficiency following siRNA-mediated depletion of Protrudin in HeLa cells.

c) WB showing the KD efficiency following siRNA-mediated depletion of RAB7 in HeLa cells.

Supplementary Figure 12

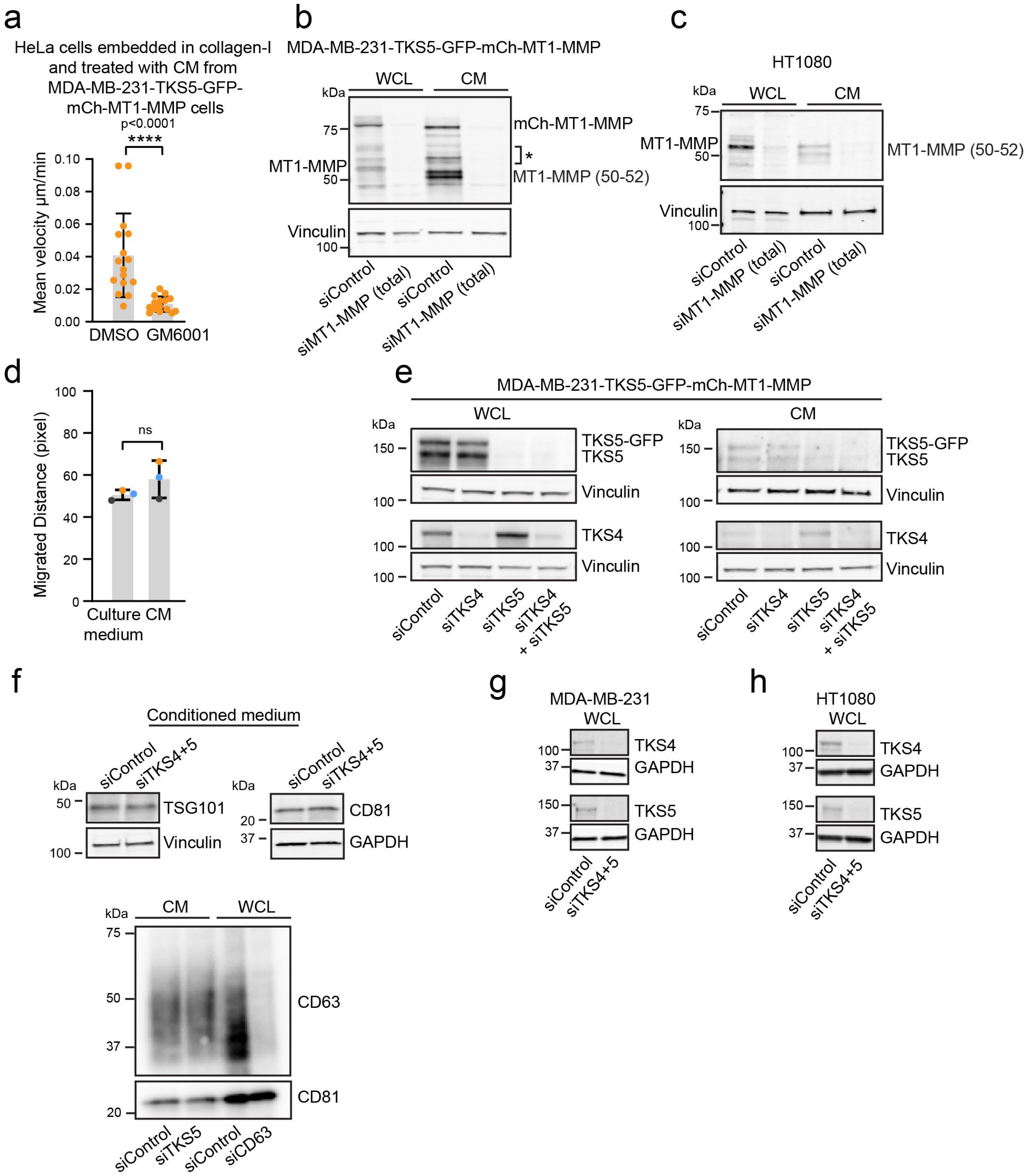

Supplementary Fig. 12 Characterization of EVs, knock down verifications and cell migration assays (related to Fig. 9)

a) HeLa cells embedded in type I collagen were incubated with conditioned medium (CM) from MDA-MB-231-TKS5-GFP-mCh-MT1-MMP cells in DMSO or 20 µM GM6001 and analysed by live cell microscopy. The graph shows the mean velocity per cell (µm min $^{-1}$). Each dot represents the mean value from one well of a multiwell imaging dish, DMSO: n=16 (269 cells), GM6001: n=15 (313 cells). Error bars denote mean +/- SD. Mann Whitney test (two-sided).

b) WB showing the KD efficiency following siRNA-mediated MT1-MMP depletion corresponding to the data described in Fig. 9a. * MT1-MMP variants likely comprising processed forms of MT1-MMP and mCh-MT1-MMP (immature, posttranslationally modified or cleaved).

c) WB showing the KD efficiency of MT1-MMP in HT1080 cells corresponding to Fig. 9b,c.

d) Random cell migration of HeLa cells treated with culture medium or CM from MDA-MB-231-TKS5-GFP-mCh-MT1-MMP cells is displayed as migrated distance (in pixel) during 24 hours of imaging. The data represents the mean +/- SD of n=3 independent experiments with 4 technical replicates each, comprising at least 3700 cells per experiment and condition. Unpaired two-sided t-test.

e) WB showing the KD efficiency following siRNA-mediated TKS4 and TKS5 depletion corresponding to the data described in Fig. 9d,e,h. Note that TKS4 is upregulated in TKS5 depleted cells.

f) Depletion of TKS4 and TKS5 does not impair exosome formation as judged by the EV markers TSG101, CD81 or CD63.

g) WB showing the KD efficiency following siRNA-mediated depletion of TKS4 and TKS5 in MDA-MB-231 cells corresponding to the data described in Fig. 9f.

h) WB showing the KD efficiency following siRNA-mediated depletion of TKS4 and TKS5 in HT1080 cells corresponding to the data described in Fig. 9g.

Supplementary Figure 13 (uncropped WB)

Fig.2c

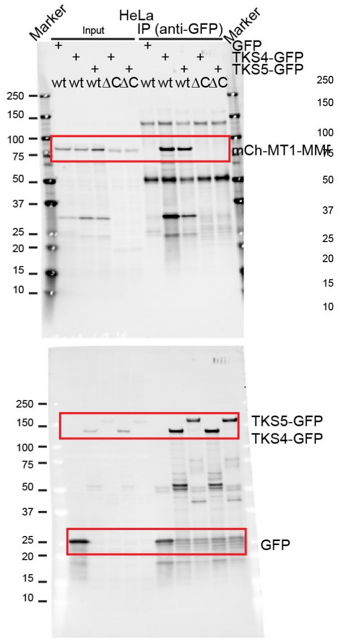

Fig.2d

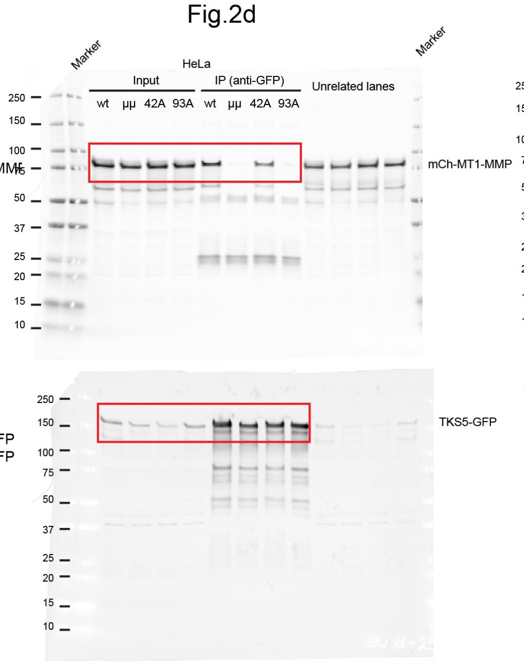

Fig.2e

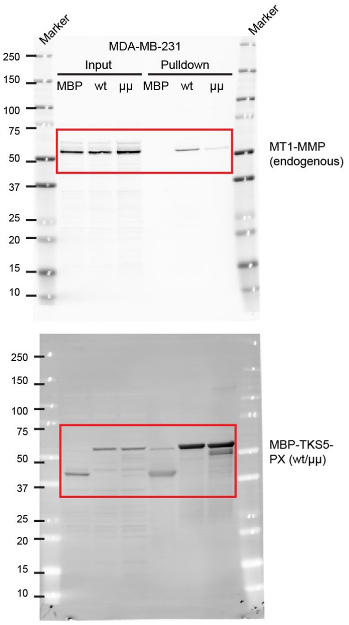

Fig.3c

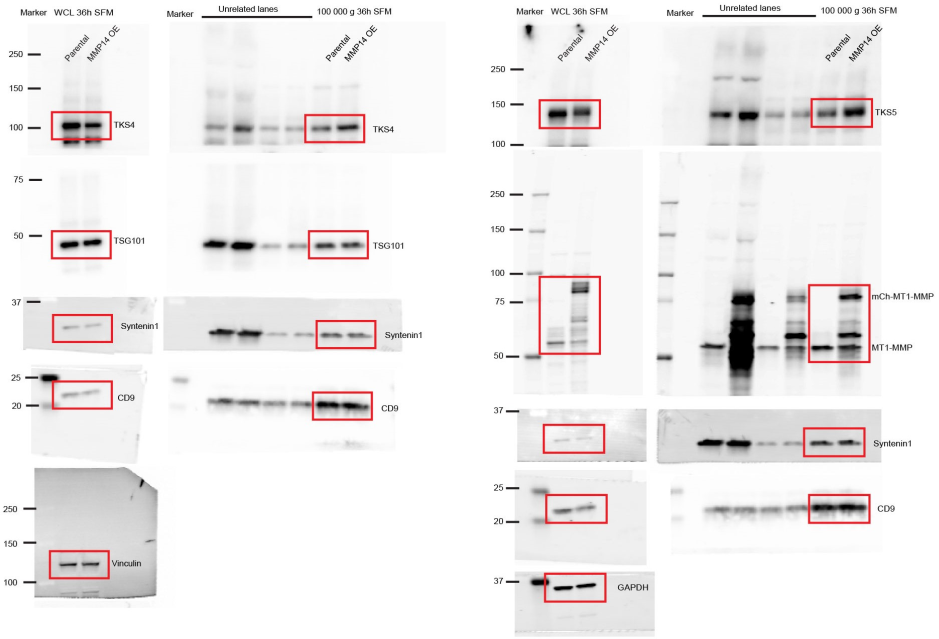

Supplementary Figure 13 (uncropped WB)

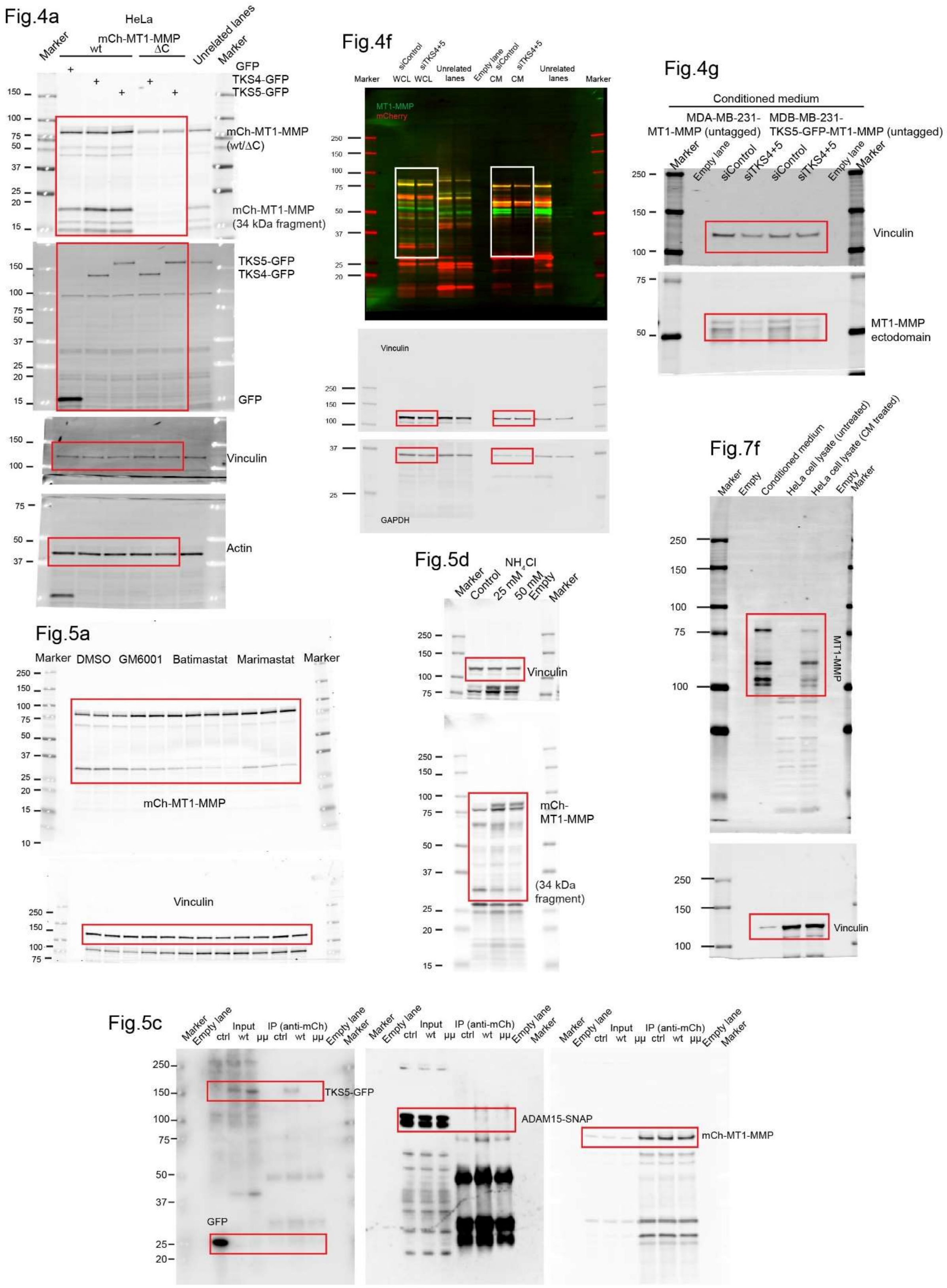

Supplementary Figure 13 (uncropped WB)

Fig.8a

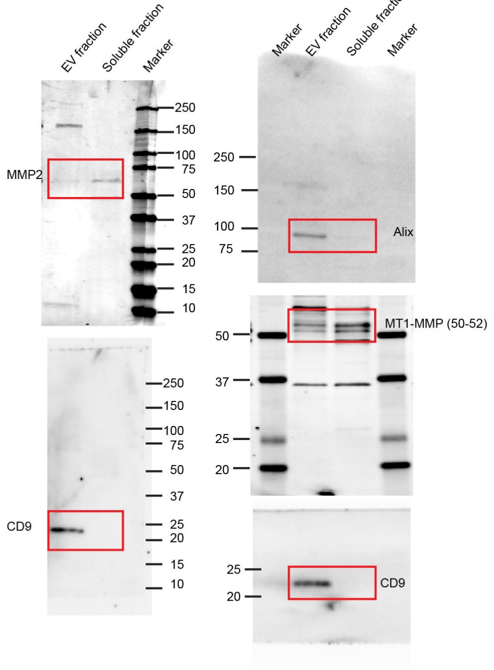

Fig.8c

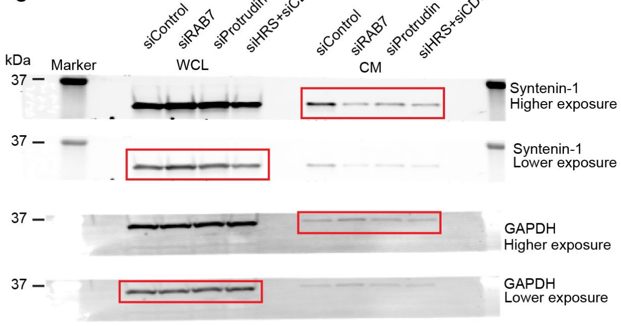

Fig.8d

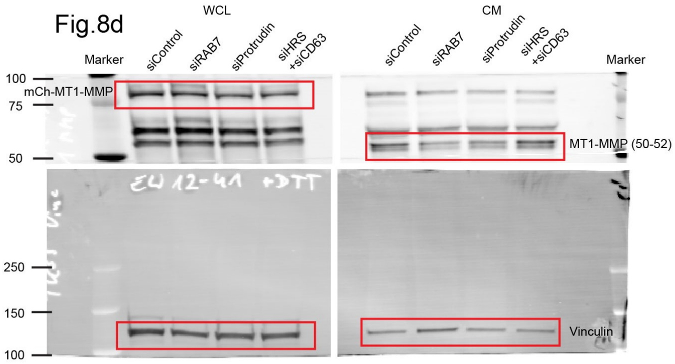

Fig.9h

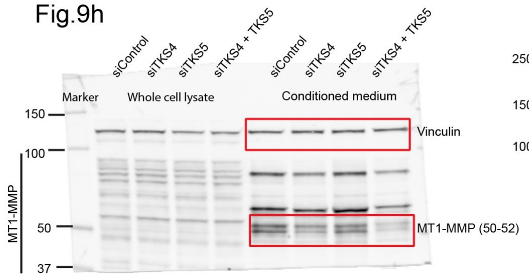

Fig.9i

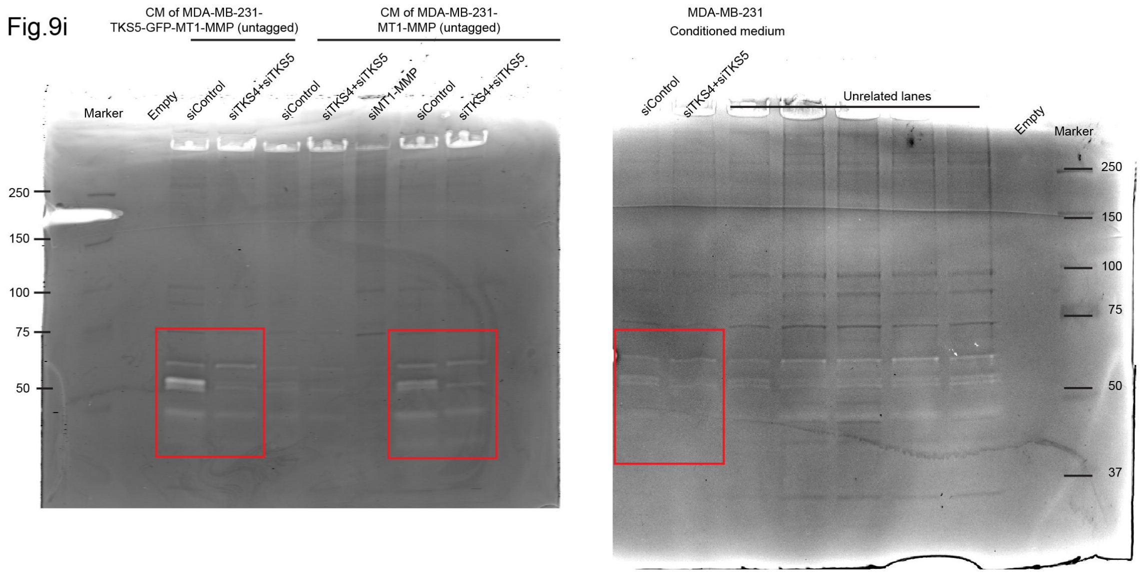

Supplementary Figure 13 (uncropped WB)

Supplementary Fig. 1c

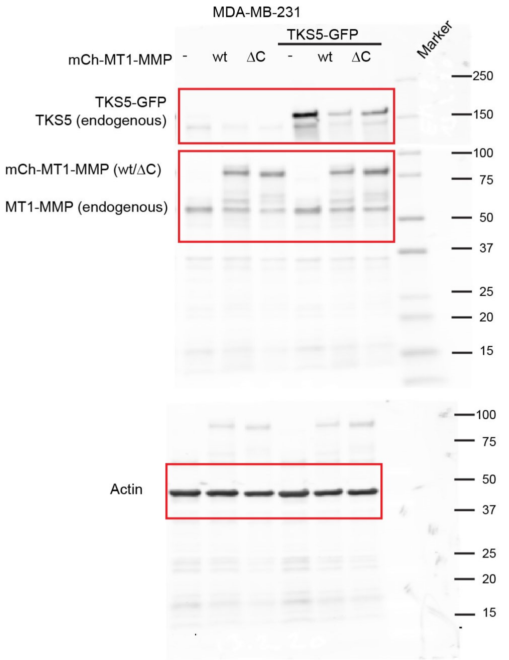

Supplementary Fig. 1e

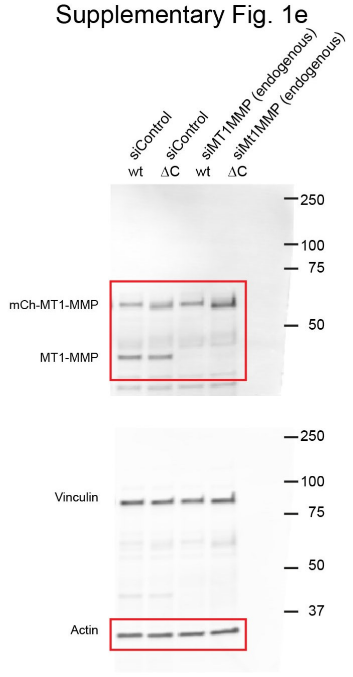

Supplementary Fig. 1h

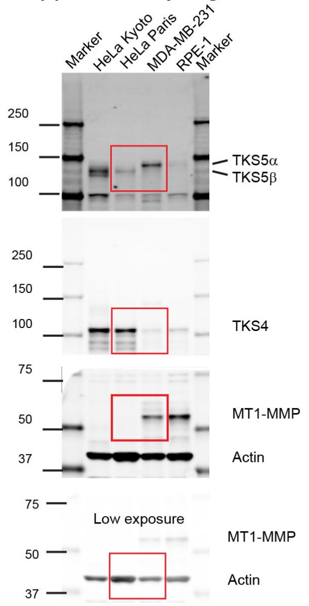

Supplementary Fig. 2a

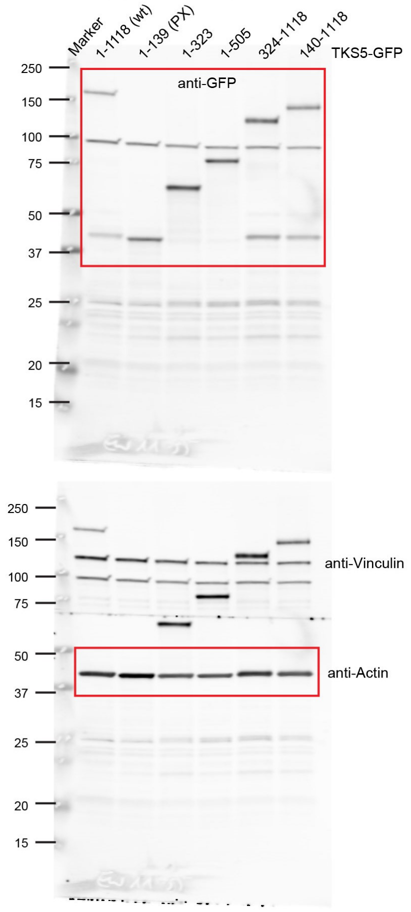

Supplementary Fig. 2c

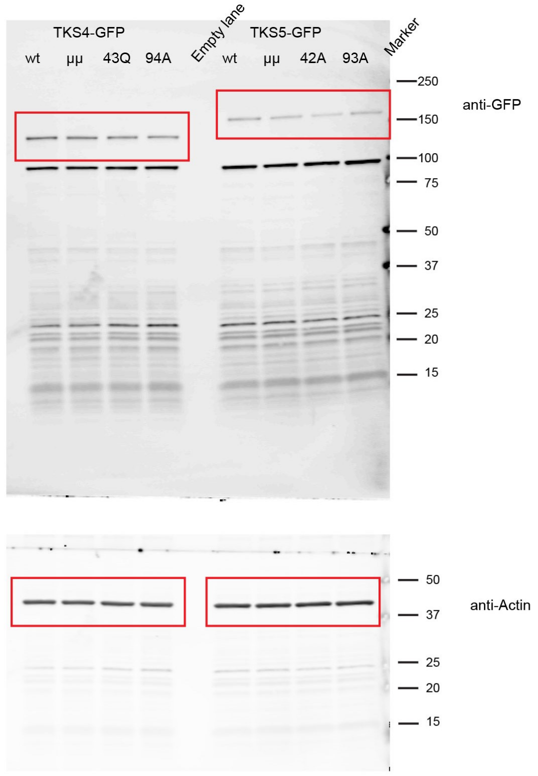

Supplementary Figure 13 (uncropped WB)

Supplementary Fig. 3a

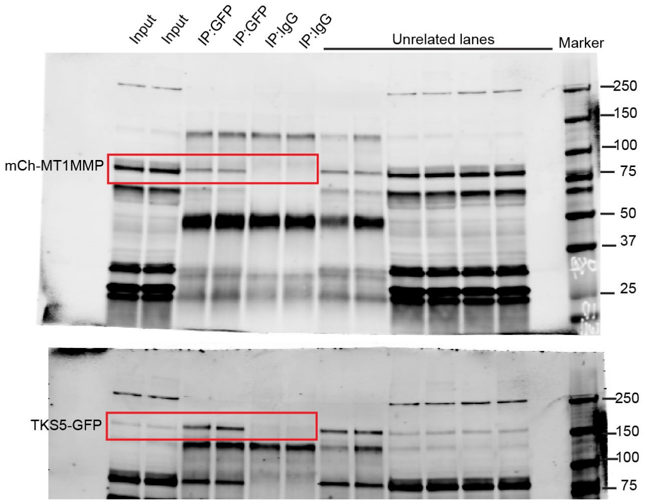

Supplementary Fig. 3b

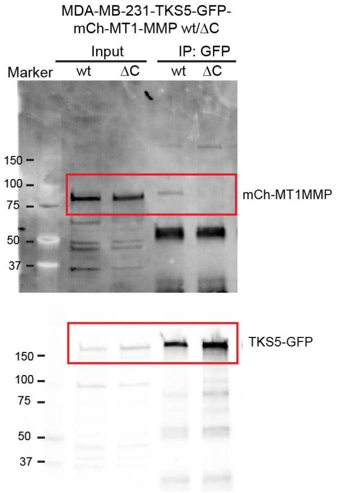

Supplementary Fig. 3c

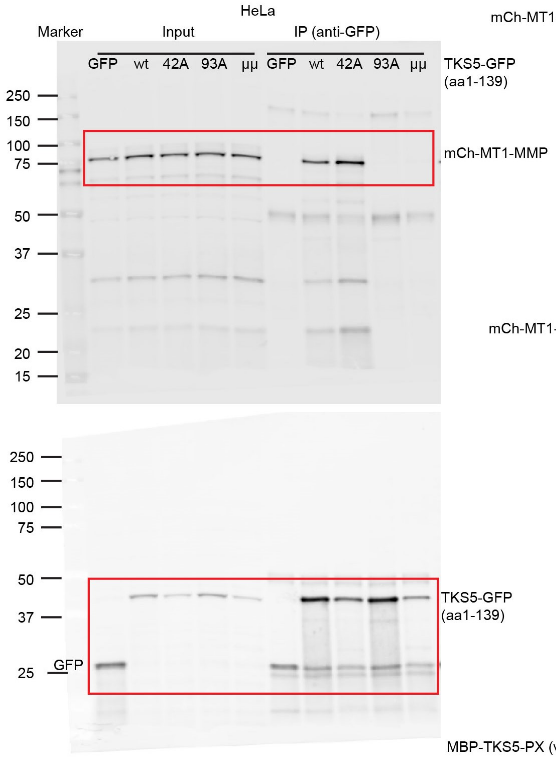

Supplementary Fig. 3d

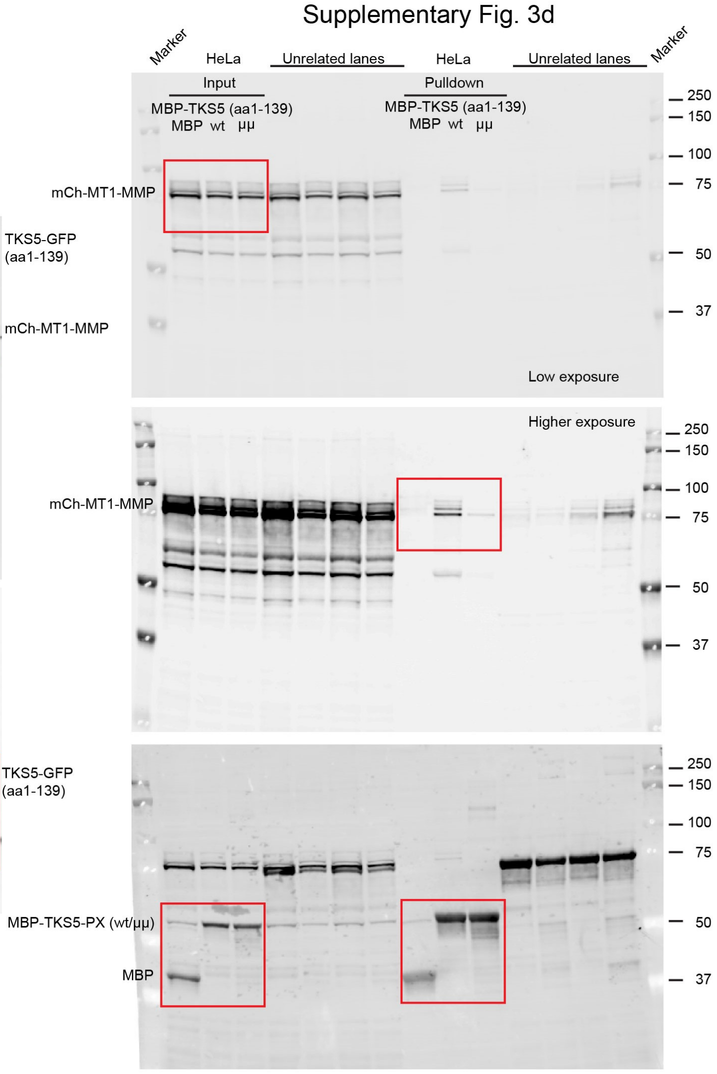

Supplementary Figure 13 (uncropped WB)

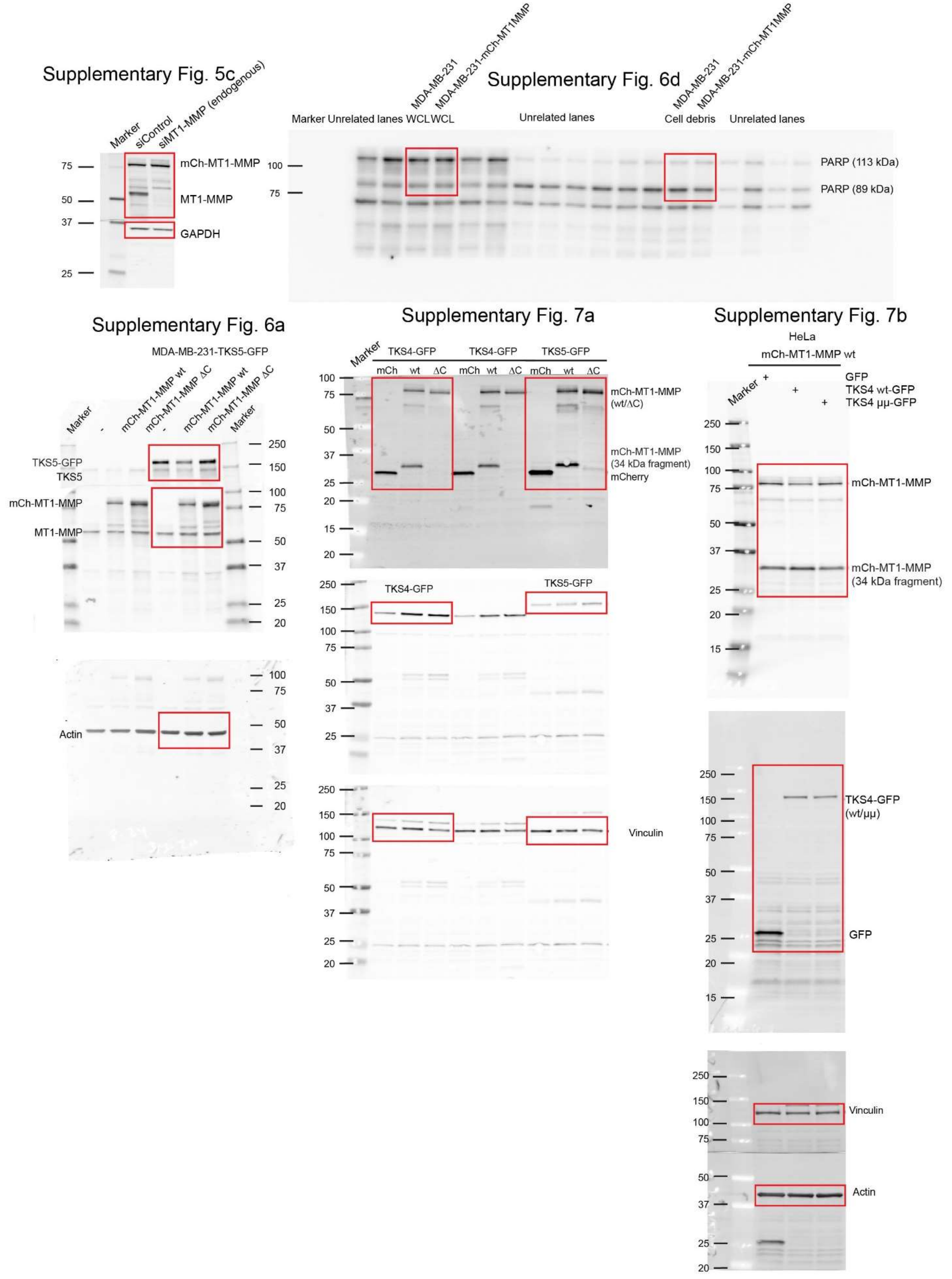

Supplementary Figure 13 (uncropped WB)

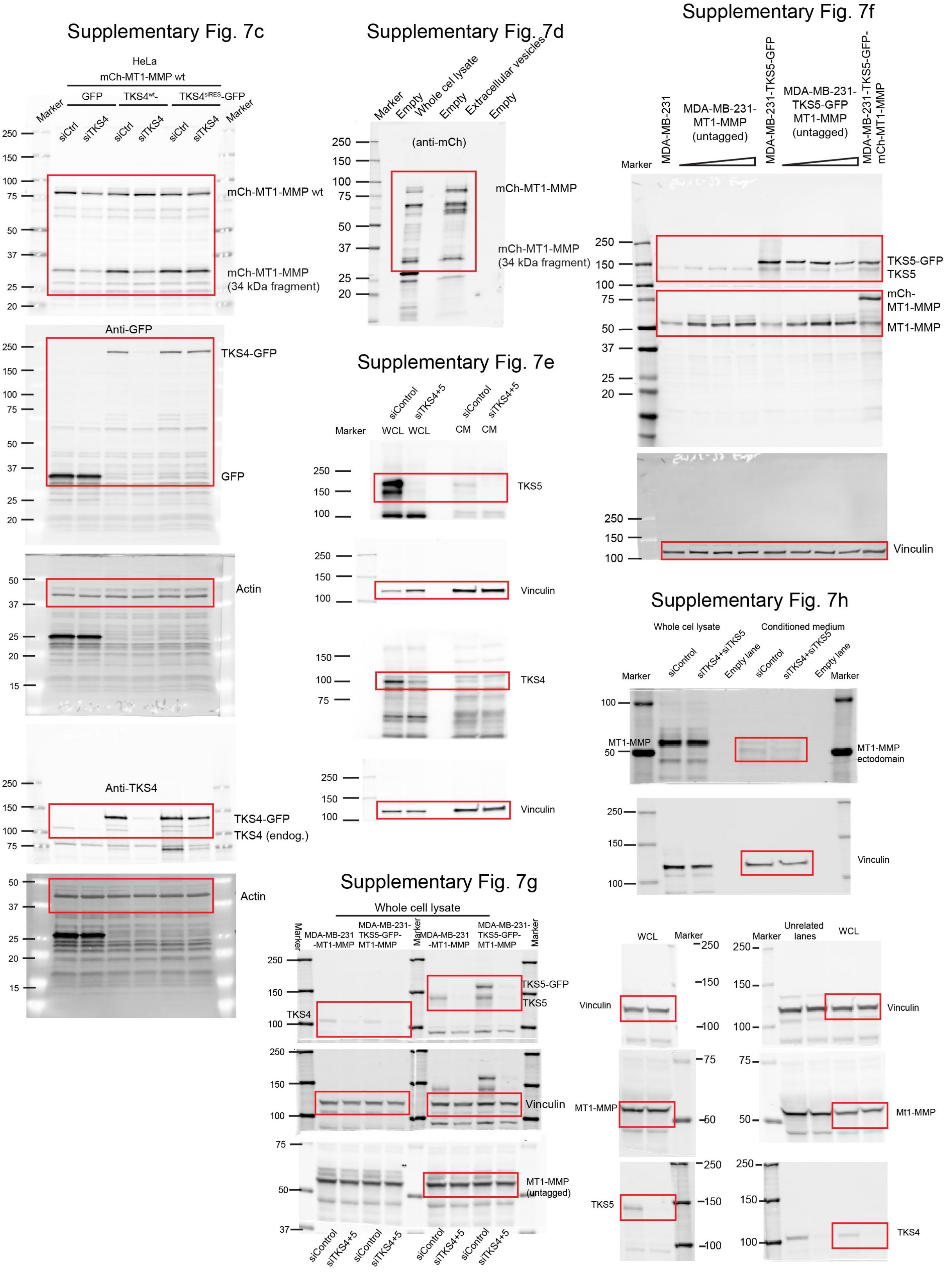

Supplementary Figure 13 (uncropped WB)

Supplementary Fig. 8a

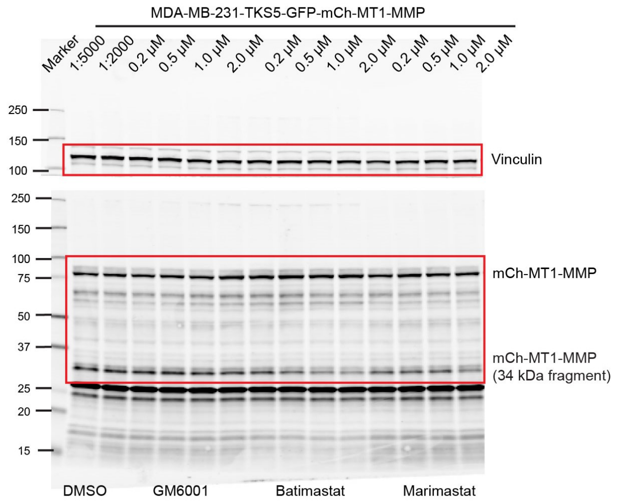

Supplementary Fig. 8d

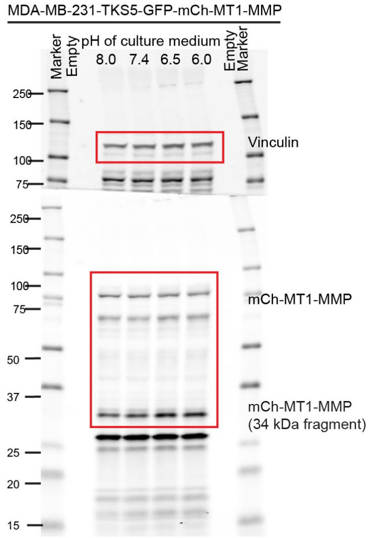

Supplementary Fig. 8c

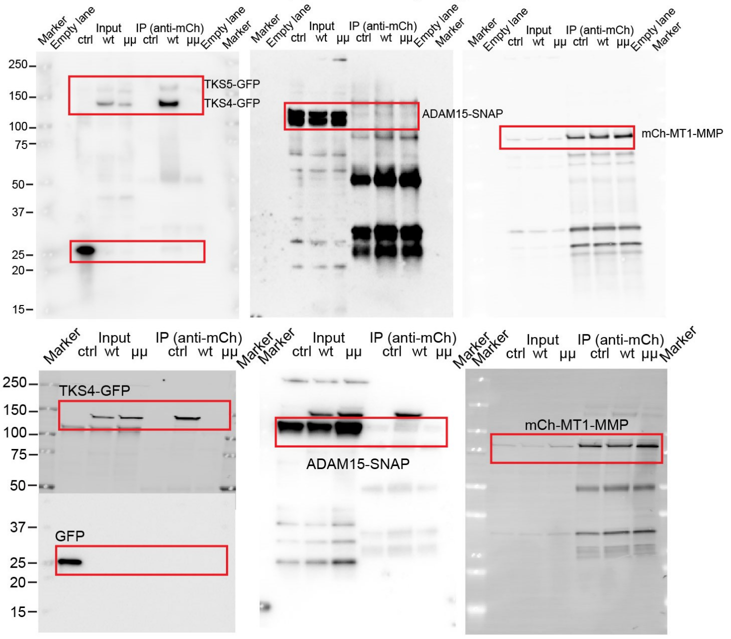

Supplementary Fig. 10e

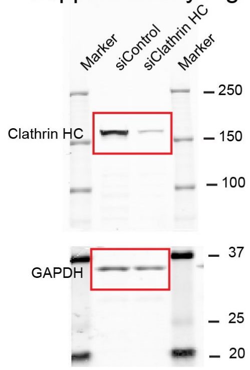

Supplementary Fig. 11b

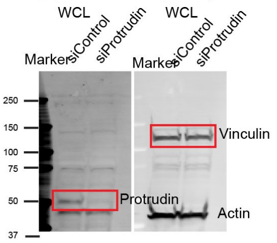

Supplementary Fig. 11a

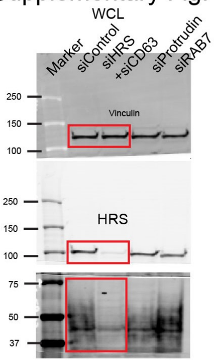

Supplementary Fig. 9a

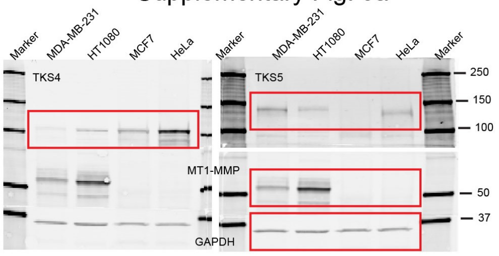

Supplementary Fig. 11c

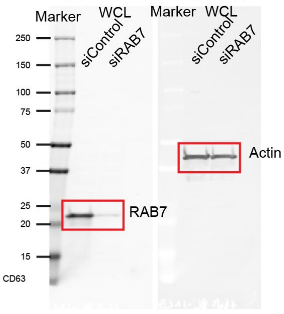

Supplementary Figure 13 (uncropped WB)

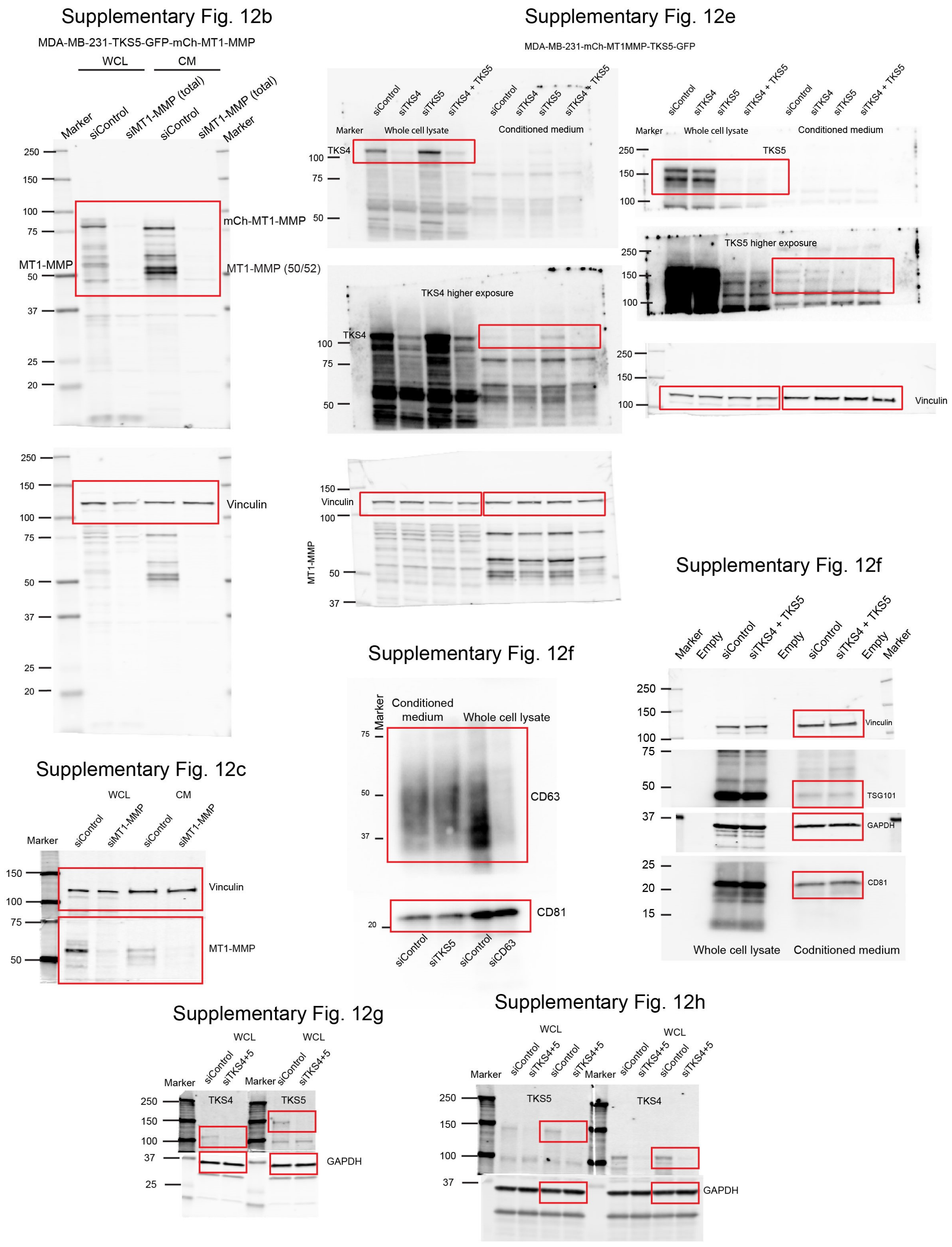

Supplementary Fig. 13 Uncropped Western blots

Uncropped Western blots from this study. The corresponding figure numbers are indicated. Red boxes outline the cropped versions shown in the original figures.
