# Supplementary material for: Intercellular transfer of cancer cell invasiveness via endosome-mediated protease shedding
Source: Nat Commun. 2024 Feb 10;15:1277. doi: 10.1038/s41467-024-45558-8 (PMC10858897; doi:10.1038/s41467-024-45558-8)
Supplement: Supplementary file 3 — Description of Additional Supplementary Files [file 41467_2024_45558_MOESM3_ESM.pdf]

Description of Additional Supplementary Files

File name: Supplementary Movie 1
Description: Live-cell imaging of MDA-MB-231-TKS5-GFP-mCh-MT1-MMP cells. DMSO (left panel) or SAR405 (right panel) were added 3 minutes after starting the image acquisition as indicated in the movie. TKS5-GFP dissociates from endosomes upon addition of SAR405. DMSO is used as a control. Corresponding movie stills are shown in Supplementary Fig. 4c.

File name: Supplementary Movie 2
Description: Live-cell imaging of MDA-MB-231-TKS5-GFP-mCh-MT1-MMP cells to illustrate that endosomal, but not invadopodial TKS5-GFP is affected by SAR405 addition. Upper panel: Maximum intensity projection of the apical part of a cell (z slices 4-33) comprising endosomes and cytosol. TKS5-GFP dissociates from endosomes upon addition of SAR405. Lower panel: Maximum intensity projection of the two most basal slices (z slices 1-2) from the image stack of the same cell, comprising invadopodia. Here, TKS5-GFP is unaffected by the addition of SAR405. Corresponding movie stills are shown in Supplementary Fig. 4d.

File name: Supplementary Movie 3
Description: Live imaging of HeLa cells embedded in collagen-I (magenta) and grown in regular culture medium (left panel) or conditioned medium from MDA-MB-231-TKS5-GFP-mCh-MT1-MMP cells (right panel). Images were acquired every 20 min for 18 hours by Nikon ECLIPSE Ti2-E microscope with a CSU-W1 confocal spinning disk. Shown is a maximum projection of z stacks. The time-lapse imaging was started 60 min after addition of medium. Note that the cells divide during the imaging. The indicated time is in h:min. Corresponding to the dataset in Fig. 6d and Supplementary Fig. 9b.
