# Supplementary material for: Intercellular transfer of cancer cell invasiveness via endosome-mediated protease shedding
Source: Nat Commun. 2024 Feb 10;15:1277. doi: 10.1038/s41467-024-45558-8 (PMC10858897; doi:10.1038/s41467-024-45558-8)
Supplement: Supplementary file 7 — Reporting Summary [file 41467_2024_45558_MOESM7_ESM.pdf]

nature portfolio

Corresponding author(s): Camilla Raiborg

Last updated by author(s): Jan 5, 2024

Reporting Summary

Nature Portfolio wishes to improve the reproducibility of the work that we publish. This form provides structure for consistency and transparency in reporting. For further information on Nature Portfolio policies, see our Editorial Policies and the Editorial Policy Checklist.

Statistics

For all statistical analyses, confirm that the following items are present in the figure legend, table legend, main text, or Methods section.

n/a	Confirmed	
☐	☒	The exact sample size (*n*) for each experimental group/condition, given as a discrete number and unit of measurement
☐	☒	A statement on whether measurements were taken from distinct samples or whether the same sample was measured repeatedly
☐	☒	The statistical test(s) used AND whether they are one- or two-sided<br>*Only common tests should be described solely by name; describe more complex techniques in the Methods section.*
☒	☐	A description of all covariates tested
☐	☒	A description of any assumptions or corrections, such as tests of normality and adjustment for multiple comparisons
☐	☒	A full description of the statistical parameters including central tendency (e.g. means) or other basic estimates (e.g. regression coefficient) AND variation (e.g. standard deviation) or associated estimates of uncertainty (e.g. confidence intervals)
☐	☒	For null hypothesis testing, the test statistic (e.g. *F*, *t*, *r*) with confidence intervals, effect sizes, degrees of freedom and *P* value noted<br>*Give P values as exact values whenever suitable.*
☒	☐	For Bayesian analysis, information on the choice of priors and Markov chain Monte Carlo settings
☒	☐	For hierarchical and complex designs, identification of the appropriate level for tests and full reporting of outcomes
☒	☐	Estimates of effect sizes (e.g. Cohen's *d*, Pearson's *r*), indicating how they were calculated

Our web collection on statistics for biologists contains articles on many of the points above.

Software and code

Policy information about availability of computer code

Data collection	Image acquisition was performed using Zen Software 2012 (Zeiss), Softworx software 7.0 (GE Healthcare), NIS elements 5.30.04 and 5.42.02 (Nikon) or Incucyte S3 (2021C).<br>WB detection was done using Odyssey software 3.0.30 (LI-COR) or AzureSpot 2.1.097 (Azure Biosystems).
Data analysis	Imaging data was analysed using ImageJ/Fiji 1.52p, Chemotaxis and Migration Tool 2.0 (IBIDI GmbH) or NIS elements 5.11 and 5.42.02 (Nikon).<br>WB quantifications were done using ImageJ/Fiji 1.52p, Odyssey software 3.0.30 (LI-COR) and Azure Spot 2.1.097 (Azure Biosystems).<br>3D image visualization was performed in Imaris 7.4.2 (Bitplane).<br>Data analysis, statistics and plotting was performed using GraphPad Prism 8.02 (Dotmatics).

For manuscripts utilizing custom algorithms or software that are central to the research but not yet described in published literature, software must be made available to editors and reviewers. We strongly encourage code deposition in a community repository (e.g. GitHub). See the Nature Portfolio guidelines for submitting code & software for further information.

Data

Policy information about availability of data

All manuscripts must include a data availability statement. This statement should provide the following information, where applicable:

- Accession codes, unique identifiers, or web links for publicly available datasets
- A description of any restrictions on data availability
- For clinical datasets or third party data, please ensure that the statement adheres to our policy

All data shown and used to generate plots, as well as detailed statistical information accompanies this manuscript in the source data file. Uncropped western blots are shown in the supplementary information. Underlying image data are available from the corresponding author upon request. Source data are provided with this paper.

Research involving human participants, their data, or biological material

Policy information about studies with human participants or human data. See also policy information about sex, gender (identity/presentation), and sexual orientation and race, ethnicity and racism.

Reporting on sex and gender	N/A
Reporting on race, ethnicity, or other socially relevant groupings	N/A
Population characteristics	N/A
Recruitment	N/A
Ethics oversight	N/A

Note that full information on the approval of the study protocol must also be provided in the manuscript.

Field-specific reporting

Please select the one below that is the best fit for your research. If you are not sure, read the appropriate sections before making your selection.

☒ Life sciences ☐ Behavioural & social sciences ☐ Ecological, evolutionary & environmental sciences

For a reference copy of the document with all sections, see nature.com/documents/nr-reporting-summary-flat.pdf

Life sciences study design

All studies must disclose on these points even when the disclosure is negative.

Sample size	No statistical methods were used to pre-determine the correct sample size. The number of experiments was adapted to the expected effect size and the anticipated consistency between experiments. All statistical calculations were derived from at least 3 biological replicates.
Data exclusions	Collected data was only excluded for technical reasons, such as unusually low transfection efficiencies or when collagen embedding for spheroid assays failed.
Replication	Quantitative data was successfully replicated in independent experiments (at least three repetitions).
Randomization	This study used cultured cell lines which are homogeneous and isogenic, therefore no randomization was used.
Blinding	Investigators were not blinded since blinding is technically and practically not feasible in this type of cell biological study. To reduce potential human bias, quantitative measurements were performed using automated image processing algorithms (using ImageJ/Fiji or NIS elements pipelines). For live cell imaging, cells were selected before their behavior was known. Key conclusions were drawn by at least two independent researchers.

Reporting for specific materials, systems and methods

We require information from authors about some types of materials, experimental systems and methods used in many studies. Here, indicate whether each material, system or method listed is relevant to your study. If you are not sure if a list item applies to your research, read the appropriate section before selecting a response.

Materials & experimental systems

n/a	Involved in the study	
☐	☒	Antibodies
☐	☒	Eukaryotic cell lines
☒	☐	Palaeontology and archaeology
☒	☐	Animals and other organisms
☒	☐	Clinical data
☒	☐	Dual use research of concern
☒	☐	Plants

Methods

n/a	Involved in the study	
☒	☐	ChIP-seq
☒	☐	Flow cytometry
☒	☐	MRI-based neuroimaging

Antibodies

Antibodies used

mouse anti-MT1-MMP (Merck/Sigma MAB3328, Lot 3074991, IF 1:200, WB 1:500)
rabbit anti-TKS5 (Merck/Sigma HPA037923, Lot D118881, IF 1:100, 1:1000-1:2000)
rabbit anti-TKS4 (Merck/Sigma HPA036471, Lot R34679, WB 1:250)
rabbit anti-TKS4 (Novus Biologicals, NBP1-93965, Lot A95495, WB 1:1000)
mouse anti-GFP (Merck/Sigma 11814460001, Lot 47859600, IF 1:500, WB 1:1000)
rabbit anti-GFP (Abcam ab6556, Lot GR3271077-1, Immuno-EM 1:500)
goat anti-mCherry (Acris AB0040-200, Lot 004018316 and 0081030119, WB 1:1000, Immuno-EM 1:50)
mouse anti-cMyc (DSHB #9E10, IF undiluted)
mouse anti-MBP (NEB, E8032S, Lot 10035197, PIP strips 1:2000)
human anti-EEA1 serum (gift from Ban-Hock Toh, Melbourne, Australia, IF 1:160000)
rabbit anti-LAMP1 (Merck/Sigma L1418, Lot 097M4836V, IF 1:400)
mouse anti-LAMP1 (DSHB #H4A3, IF 1:400)
mouse anti-CD63 (DSHB #H5C6, WB 1:500, Immuno-EM 1:50)
mouse anti-CD81 (Ancell Corporation 302-820, Lot 173603/254702, WB 1:500)
rabbit anti-CD9 (Abcam, ab92726, Lot GR3252550-10, WB 1:1000)
rabbit anti-CD9 (Abcam, ab263019, Lot GR3395447-22, WB 1:1000)
mouse anti-TSG101 (BD Transduction Lab 612697, Lot 7153736, WB 1:500)
mouse anti-cortactin (Merck/Sigma 05–180, Lot 2967772, IF 1:200)
rabbit anti-PARP1 (Cell signalling technologies 9542, Lot 15, WB 1:500)
rabbit anti-Syntenin-1 (Abcam, ab133267, Lot GR3375272-9,WB 1:1000)
rabbit anti-clathrin heavy chain (Abcam, ab21679, Lot GR179225-1, WB 1:1000)
rabbit anti-HRS serum (rabbits were immunized with MBP-HRS. Described in Raiborg et al. 2001 EMBO J. WB 1:1000)
rabbit anti-Alix (rabbits were immunized with MBP-Alix. Described in Cabezas et al. 2005 JCS, WB 1:3000)
mouse anti-MMP2 (Calbiochem, #IM33, clone 42-5D11, Lot. D00008295, WB 1:1000)
rabbit anti-Zyxin (HPA004835, Lot A38795, IF 1:100)
mouse anti-β-actin (Merck/Sigma A5316, Lot 096M4855V, WB 1:5000)
mouse anti-vinculin (Merck/Sigma V9131, Lot 0000128055, WB 1:3000)
mouse anti-GAPDH (Abcam, ab9484, Lot GR3238413-1, WB 1:3000)

Validation

mouse anti-MT1-MMP (Merck/Sigma MAB3328), rabbit anti-TKS5 (Merck/Sigma HPA037923, Lot D118881), rabbit anti-TKS4 (Merck/Sigma HPA036471), rabbit anti-TKS4 antibody (Novus Biologicals, NBP1-93965), goat anti-mCherry (Acris AB0040-200), mouse anti-CD63 (DSHB #H5C6), rabbit anti-CHC (Abcam, ab21679), rabbit anti-HRS (selfmade) were validated by KD experiments in this manuscript.

The following antibodies have been previously used and published in our lab (Raiborg et al. 2002, Bache et al. 2006, Phuyal et al. 2014, Hong et al. 2017, Wenzel et al. 2018, Pedersen et al. 2020, Hessvik et al. 2023, Cabezas et al. 2005) and by others:
mouse anti-GFP (Merck/Sigma 11814460001): applications: IF, WB, IP. Cited in more than 700 publications according to Merck.
mouse anti-β-actin (Merck/Sigma A5316): applications: IF, WB. Cited in more than 4100 publications according to citeab.com.
mouse anti-vinculin (Merck/Sigma V9131): applications: IF, WB. Cited in more than 1600 publications according to Merck.
mouse anti-GAPDH antibody (Abcam, ab9484): application: WB. Cited in more than 700 publications according to Abcam.
mouse anti-cMyc (DSHB #9E10): applications: IF, WB. Cited in 51 publications according to DSHB.
mouse anti-LAMP1 antibody (DSHB #H4A3): applications: IF, WB. Cited in 39 publications according to DSHB.
mouse anti-CD63 antibody (DSHB #H5C6): applications: IF, WB. Cited in 22 publications according to DSHB.
human anti-EEA1 serum (gift from Ban-Hock Toh, Melbourne, Australia). Described in Mu et al. 1995 JCB.
rabbit anti-LAMP1 (Merck/Sigma L1418): applications: IF, WB. Cited in 94 publications according to Merck.
mouse anti-CD81 antibody (Ancell Corporation 302-820): application: WB. Cited in 9 publications according to ancell.com.
rabbit anti-CD9 antibody (Abcam, ab92726): applications: WB, IP. Cited in 607 publications according to citeab.com.
mouse anti-TSG101 (BD Transduction Lab 612697): application: WB. Cited in 15 publications according to citeab.com.
mouse anti-cortactin (Merck/Sigma 05–180): applications: WB, IF. Cited in 277 publications according to citeab.com.
rabbit anti-GFP (Abcam ab6556): applications: EM, IP, WB. In total more than 1000 references according to Abcam.
rabbit anti-PARP1 antibody (Cell signalling technologies 9542, Lot 15, WB 1:500): applications: IF, WB. Cited in more than 10000 publications according to Cell signalling technolgies.
rabbit anti-Syntenin-1 (Abcam, ab133267): applications: IF, WB. Cited in 95 publications according to citeab.com.
rabbit anti-Alix (selfmade, validated by KD for WB in Cabezas et al. 2005 JCS)

The following antibody has been used and published by other labs:
mouse anti-MBP antibody (NEB, E8032S, Lot 10035197): applications: WB, ELISA. Cited in more than 700 publications according to NEB.

	rabbit anti-Zyxin (HPA004835, Lot A38795, IF 1:100): appliclations: IF. Cited in 6 publications according to citeab.com.<br>mouse anti-MMP2 (Calbiochem #IMM): applications: WB. Cited in 111 publications according to citeab.com.

Eukaryotic cell lines

Policy information about cell lines and Sex and Gender in Research

Cell line source(s)	MDA-MB-231, MCF7 and HT1080 cells were purchased from ATCC. HeLa cells were obtained from Institute Curie, Paris, France.
Authentication	Cell lines were authenticated by the Genotyping core facility at Oslo University Hospital using Powerplex16 assays.
Mycoplasma contamination	Cell lines were regularly tested for mycoplasma contamination. All cell lines were negative for mycoplasma infection.
Commonly misidentified lines<br>(See ICLAC register)	none

Plants

Seed stocks	N/A
Novel plant genotypes	N/A
Authentication	N/A
